# Nanotechnology-enhanced immunotherapies for pancreatic ductal adenocarcinoma: challenges and opportunities

**DOI:** 10.1007/s13346-025-01908-7

**Published:** 2025-07-08

**Authors:** Sheng Yang, Yen-Nhi Ngoc Ta, Yunching Chen

**Affiliations:** 1 Institute of Biomedical Engineering, National Tsing Hua University, Hsinchu, 30013 Taiwan; 2 International Intercollegiate PhD Program, National Tsing Hua University, Hsinchu, 30013 Taiwan; 3 Department of Chemistry, National Tsing Hua University, Hsinchu, 30013 Taiwan

**Keywords:** Pancreatic ductal adenocarcinoma (PDAC), Tumor microenvironment, Desmoplasia, Vessel normalization, Cancer vaccine

## Abstract

Pancreatic ductal adenocarcinoma (PDAC) is a highly aggressive malignancy with a dismal five-year survival rate. The tumor microenvironment, characterized by dense stroma and immunosuppression, significantly limits the effectiveness of current treatments, including immunotherapy. Nanotechnology offers a promising solution by enabling precise drug delivery, enhancing immune activation, and overcoming physical barriers within the tumor microenvironment. In this review, we explore the mechanisms of PDAC development and the immunosuppressive nature of its tumor microenvironment, emphasizing the challenges faced by immunotherapy. We also examine the role of nanotechnology in modulating the tumor microenvironment, enhancing cancer vaccine efficacy, and stimulating immune cells within the PDAC tumor microenvironment. Current clinical trials employing nanotechnology-based strategies are discussed, providing insight into their potential for improving therapeutic outcomes. While significant challenges remain in translating nanomedicine into clinical success, the integration of nanotechnology with immunotherapy holds promise for overcoming the limitations of conventional treatments and improving survival in PDAC patients.

**Graphical Abstract:**

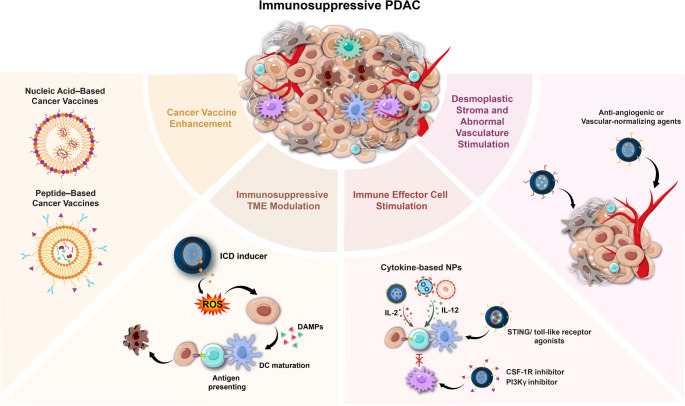

## Introduction

Pancreatic ductal adenocarcinoma (PDAC), the most common form of pancreatic cancer, accounts for over 90% of cases [1]. According to estimates from the National Cancer Institute, PDAC represents only 3.3% of all new cancer cases—ranking tenth—but is responsible for 8.4% of all cancer-related deaths, making it the third leading cause of cancer mortality in the United States. Despite ongoing advancements in diagnostic tools and treatment modalities, the overall prognosis remains bleak, with a five-year survival rate lingering between 10% and 13% [2]. This poor outcome is primarily due to late-stage diagnosis, rapid disease progression, and the limited effectiveness of current standard-of-care therapies [3, 4]. At the time of presentation, fewer than 20% of patients qualify for potentially curative surgical resection [5, 6]. The majority are diagnosed with either locally advanced or metastatic disease.

Treatment approaches are largely dictated by the disease stage. Patients with resectable PDAC are generally treated with surgery followed by adjuvant chemotherapy—most often with modified FOLFIRINOX (mFOLFIRINOX). FOLFIRINOX is a chemotherapy regimen made up of leucovorin calcium (folinic acid), fluorouracil, irinotecan hydrochloride, and oxaliplatin. In view of its high toxicities, the dosage of mFOLFIRINOX is adjusted, showing similar efficacy but better safety. mFOLFIRINOX demonstrates improved survival outcomes compared to gemcitabine [1, 7, 8]. In borderline resectable or locally advanced cases, neoadjuvant therapies are employed to enable surgical resection [9]. For metastatic PDAC, systemic chemotherapy remains the mainstay, with regimens such as FOLFIRINOX or gemcitabine plus nab-paclitaxel (nab-PTX) providing limited survival benefits [10]. However, treatment-related toxicity poses a significant challenge, especially for older or frail individuals. Targeted therapies and immune checkpoint inhibitors (ICIs) have shown promise in a subset of patients with specific biomarkers, such as BRCA mutations—a breast cancer gene—or microsatellite instability [11], but the majority of patients do not benefit from these therapies.

A major barrier to therapeutic success in PDAC is its dense, immunosuppressive tumor microenvironment. The tumor microenvironment is characterized by extensive desmoplasia and a dense extracellular matrix (ECM), which may comprise up to 80% of the tumor volume. These physical features hinder vascular perfusion, drug penetration, and immune cell infiltration [12, 13]. Concurrently, the immune compartment is dominated by suppressive myeloid cells, such as tumor-associated macrophages (TAMs), myeloid-derived suppressor cells (MDSCs), and regulatory T cells (Tregs), with a marked deficiency in cytotoxic T lymphocytes [3, 14]. These factors render PDAC immunologically “cold” and unresponsive to ICIs and adoptive cell therapies—approaches that have otherwise demonstrated success in melanoma, lung cancer, and hematologic malignancies.

Against this backdrop, nanotechnology offers a transformative opportunity to surmount the inherent barriers of PDAC and enhance the effectiveness of immunotherapies. Engineered nanoparticles (NPs) can improve the pharmacokinetics and intratumoral retention of immunotherapeutic agents, facilitate the co-delivery of synergistic payloads (such as antigens, adjuvants, cytokines, or checkpoint inhibitors), and provide spatial and temporal control over immune activation [15]. Additionally, NPs can be tailored to penetrate fibrotic stroma, reprogram immunosuppressive cellular components within the tumor microenvironment, and reduce systemic toxicity. These properties uniquely position nanomedicine as a promising platform for converting PDAC from an immune-refractory tumor into one that is amenable to immunologic intervention.

This review provides a comprehensive overview of the clinical and immunological landscape of PDAC, emphasizing the role of the tumor microenvironment in immune evasion and therapeutic resistance. We discuss emerging strategies that leverage nanotechnology to reprogram the tumor microenvironment, enhance immune priming, and enable synergistic combination immunotherapies. Finally, we highlight recent clinical developments and future directions for incorporating nanomedicine into the evolving treatment paradigm of PDAC.

## The role of the tumor microenvironment in PDAC progression and immunosuppressive challenges

The tumor microenvironment of PDAC refers to the dynamic and heterogeneous ecosystem surrounding tumor cells, comprising a complex interplay of cellular, extracellular, and molecular components that collectively shape disease progression, immune evasion, and therapeutic resistance. This microenvironment includes not only neoplastic pancreatic cells but also stromal elements such as cancer-associated fibroblasts (CAFs), pancreatic stellate cells (PSCs), immune infiltrates (e.g., MDSCs, TAMs, regulatory and effector T cells), endothelial cells, ECM proteins, and aberrant vasculature. In PDAC, this tumor microenvironment is uniquely characterized by dense desmoplasia, poor vascularization, hypoxia, and profound immunosuppression.

These components work in concert to create a tumor-favorable niche. CAFs, for instance, drive extensive fibrosis and ECM deposition, forming a physical barrier that limits drug delivery and immune cell infiltration. MDSCs and TAMs suppress antitumor immunity by inhibiting cytotoxic T cells and promoting regulatory T cell expansion. Additionally, abnormal vasculature, altered neural signaling, and matrix metalloproteinases (MMPs) contribute to tumor invasion and metastasis [16]. Collectively, the PDAC tumor microenvironment not only facilitates tumor progression but also poses a major obstacle to therapeutic efficacy—especially for immunotherapies. Therefore, dissecting the cellular and molecular composition of the tumor microenvironment is critical for designing strategies that can effectively reprogram this hostile landscape and improve treatment outcomes.

### PDAC driven by genes and microenvironment

Pancreatic ductal adenocarcinoma (PDAC) develops through a well-defined multistep process involving the transition from noninvasive precursor lesions to invasive carcinoma. The three primary precursor lesions—pancreatic intraepithelial neoplasia (PanIN), intraductal papillary mucinous neoplasms (IPMNs), and mucinous cystic neoplasms (MCNs) [12, 17]—differ in morphology and molecular features but converge on a common endpoint of malignant transformation. PanINs are the most prevalent and microscopic in nature, while IPMNs and MCNs are cystic and more easily detectable radiologically. Progression from low-grade to high-grade lesions is accompanied by the stepwise accumulation of genetic alterations that enable the transformation of normal epithelium into invasive cancer. Although early detection and surgical resection of high-grade precursors offer a potential window for cure, most PDACs are diagnosed after the disease has become invasive and metastatic.

The genetic landscape of PDAC is characterized by a core set of driver mutations that orchestrate tumor initiation and progression. Activating mutations in KRAS are present in over 90% of PDAC cases and represent the earliest and most common oncogenic event. Constitutively active KRAS drives aberrant signaling through the MAPK and PI3K pathways, supporting proliferation, metabolic reprogramming, and survival [12]. Inactivation of CDKN2A, which encodes the tumor suppressor p16^INK4A^, is also an early event and facilitates uncontrolled cell cycle progression by disabling the G1–S checkpoint. As the disease advances, mutations in TP53 and SMAD4 become prevalent. Loss of p53 function promotes genomic instability and resistance to apoptosis, while SMAD4 inactivation disrupts TGF-β–mediated tumor suppression and is strongly associated with metastasis [3, 18]. Together, these four genes—KRAS, CDKN2A, TP53, and SMAD4—constitute the central molecular framework of PDAC. Their collective impact confers hallmark capabilities including sustained growth, resistance to cell death, and metastatic potential.

Additional alterations in “hill” genes, such as BRCA2, RNF43, PALB2, and TGFBR2, occur in a subset of cases and may inform personalized treatment strategies [19]. Importantly, the timeline from the first oncogenic mutation to the emergence of invasive PDAC spans several years, followed by more rapid metastatic spread. This prolonged latency contrasts with the typically late clinical presentation of PDAC, highlighting a critical gap between biological development and clinical detectability.

The progression of PDAC is not governed solely by genetic mutations; it is profoundly shaped by the co-evolving tumor microenvironment. A defining feature of PDAC is its extensive desmoplastic stroma, comprising ECM components and a heterogeneous population of non-neoplastic cells. The bulk of this fibrotic reaction is driven by CAFs, many of which originate from PSCs [20]. PSCs are star-shaped cells located in the pancreas, expressing specific markers such as desmin, nestin, α-SMA, and glial fibrillary acidic protein (GFAP). In their quiescent state, PSCs store vitamin A in lipid droplets and maintain pancreatic homeostasis, modulate the ECM, and facilitate wound repair. However, under the influence of tumor-secreted factors such as TGF-β, IL-1, and Hedgehog ligands, quiescent PSCs are activated and adopt a myofibroblastic phenotype, marked by α-smooth muscle actin (α-SMA) expression and secretion of collagen and fibronectin, thus contributing to the physical density and hypovascularity of PDAC tumors [21].

Recent studies have revealed that cancer-associated fibroblasts (CAFs) are a heterogeneous population comprising distinct subtypes with specialized and sometimes opposing functions. Among them, the most well-characterized subtypes are inflammatory CAFs (iCAFs), myofibroblastic CAFs (myCAFs), and antigen-presenting CAFs (apCAFs) (Table 1). iCAFs primarily originate from CD105⁻ fibroblast populations and are typically located at a distance from tumor cells. Their activation is driven by the IL-1/JAK/STAT signaling axis, leading to the secretion of pro-inflammatory cytokines such as IL-6 and leukemia inhibitory factor (LIF). These cytokines promote tumor proliferation, resistance to apoptosis and chemotherapy, and contribute to the formation of an immunologically “cold” tumor microenvironment. iCAFs also recruit immunosuppressive cells—including myeloid-derived suppressor cells (MDSCs) and regulatory T cells (Tregs)—and secrete CXCL12 and extracellular matrix (ECM) components that hinder T cell infiltration. Furthermore, iCAFs express high levels of HAS1 and HAS2, enzymes involved in hyaluronan synthesis, which enhance ECM density and further restrict immune cell access to the tumor [4, 20–24].

**Table 1:** The markers and functions of CAF subtypes

CAF subtype	Resource	Resident	Markers	Activator	Function	Ref
**iCAF**	CD105^neg^ fibroblasts	Located in proximity to tumor cells	CXCL1, CXCL12, AGTR1, Has1, Has2, IL6, LIF, PDGFRα	IL-1	Facilitating tumor proliferation; enhancing ECM formation; recruiting immunosuppressive cells into tumor microenvironment	[20–24]
**myCAF**	CD105^pos^ fibroblasts	Located distally to tumor cells	ACTA2 (α-SMA), TAGLN, MYL9 and MMP11, LRRC15	TGF-β	Promoting ECM formation; increasing interstitial pressure	[20–25]
**apCAF**	Mesothelial cells		H2-Ab1, MHC II (CD74), Saa3	IL-1, TGF-β	Inducing Treg expansion	[20, 24, 26, 27]

In contrast, myCAFs are located in close proximity to tumor cells and arise from CD105⁺ fibroblast populations. They express high levels of α-smooth muscle actin (α-SMA) and low levels of inflammatory cytokines. myCAFs are primarily involved in ECM production and tissue remodeling, with their activation strongly influenced by TGF-β signaling, which induces the expression of contractile proteins such as TAGLN, MYL9, and MMP11. The dense stroma formed by myCAFs contributes to elevated interstitial pressure and limited immune cell infiltration, thereby promoting an immunosuppressive microenvironment [22, 24, 25]. Importantly, iCAFs and myCAFs exhibit a high degree of plasticity; TGF-β favors myCAF differentiation, while IL-1α promotes the iCAF phenotype, allowing dynamic shifts in CAF subtypes based on local cytokine cues. Interestingly, therapeutic attempts to deplete CAFs entirely have sometimes led to more aggressive tumor phenotypes in preclinical models, highlighting the dualistic and context-dependent roles of the stroma. Rather than total depletion, modulating the iCAF/myCAF ratio to reduce pro-tumor cytokine secretion and enhance anti-tumor immunity has emerged as a promising strategy in PDAC.

A third, less abundant subtype, antigen-presenting CAFs (apCAFs), likely originates from mesothelial cells, transitioning through pathways regulated by both IL-1/NF-κB and TGF-β/SMAD signaling [26]. apCAFs are characterized by expression of MHC class II molecules such as CD47 and H2-Ab1, enabling them to present antigens to CD4⁺ T cells. However, in the absence of co-stimulatory signals, apCAFs may contribute to immune tolerance by promoting Treg expansion, further shaping the immunosuppressive tumor microenvironment [20, 24, 26, 27].

Tumor cells and stromal components engage in constant reciprocal signaling that reinforces malignancy. Tumor-derived factors activate CAFs and other stromal cells, which in turn secrete growth factors, cytokines, and chemokines that enhance cancer cell survival, invasiveness, and immune evasion. This feedback loop creates a self-sustaining ecosystem in which both genetic mutations and stromal remodeling work in concert to support tumor progression and therapeutic resistance.

In summary, PDAC is a paradigmatic example of a malignancy driven by both intrinsic genetic alterations and an extrinsically hostile microenvironment. The co-evolution of tumor cells and the stroma creates a layered defense system that protects the tumor from immune attack and therapeutic intervention. As such, future therapeutic strategies must address both compartments—targeting driver mutations and reprogramming the tumor microenvironment—to dismantle this formidable tumor ecosystem [28]. Understanding the molecular and cellular mechanisms underpinning this interplay is essential for the development of effective, multifaceted treatment approaches.

### The PDAC tumor microenvironment as a barrier to immunotherapy

Immunotherapy refers to a range of strategies that activate, redirect, or enhance the host immune system to recognize and eliminate cancer cells. While ICIs, adoptive cell transfer, and cancer vaccines have revolutionized the treatment of melanoma, non-small cell lung cancer, and other malignancies, these successes have not yet translated into meaningful clinical benefit for patients with pancreatic ductal adenocarcinoma (PDAC) [29]. A defining barrier to immunotherapy efficacy in PDAC is its profoundly immunosuppressive and physically restrictive tumor microenvironment, which prevents immune activation at multiple levels.

**Fig. 1:**
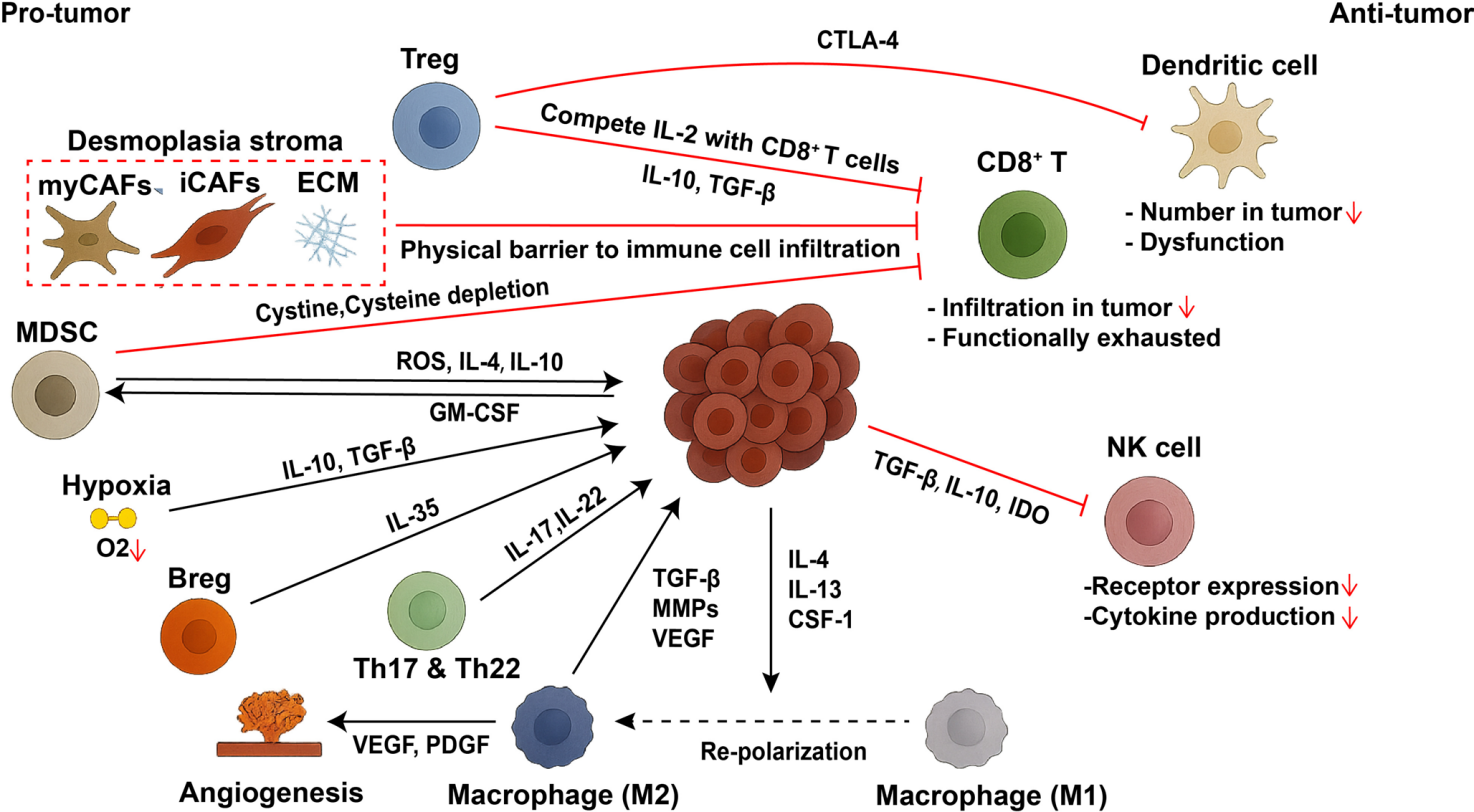
Immunosuppressive networks within the PDAC tumor microenvironment. The PDAC tumor microenvironment is dominated by dense desmoplastic stroma composed of extracellular matrix (ECM), cancer-associated fibroblasts (CAFs), and activated pancreatic stellate cells (aPSCs), forming a physical barrier that impedes immune cell infiltration. Hypoxia within the stroma induces the secretion of immunosuppressive cytokines such as IL-10 and TGF-β, further promoting immune tolerance. Tumor-secreted GM-CSF recruits myeloid-derived suppressor cells (MDSCs), which inhibit T cell activation through cystine/cysteine depletion, ROS production, and immunosuppressive cytokine secretion. Tumor-associated macrophages (TAMs), polarized to an M2 phenotype by IL-4, IL-13, and CSF-1, secrete TGF-β, VEGF, and MMPs, thereby suppressing effector T cell activity and supporting angiogenesis and tissue remodeling. Dendritic cells (DCs) are diminished and functionally impaired, limiting effective T cell priming. Natural killer (NK) cells are reduced in number and function, with decreased activating receptor expression and cytokine production. CD8⁺ cytotoxic T cells are scarce and functionally exhausted, while regulatory T cells (Tregs) suppress effector T cell responses through IL-10, TGF-β, IL-2 competition, and CTLA-4-mediated inhibition. Additional suppressive populations, including Th17, Th22, and Breg cells, contribute to the establishment of an immunosuppressive, tumor-permissive microenvironment that limits the efficacy of immunotherapies

The PDAC tumor microenvironment is dominated by a dense desmoplastic stroma, composed of ECM, CAFs, and activated pancreatic stellate cells (aPSCs) (Fig. 1). This fibrotic barrier compresses blood vessels, limits perfusion, and physically excludes immune effector cells such as cytotoxic T lymphocytes (CTLs) and natural killer cells (NK cells) from accessing tumor cells. Compounding this structural challenge, the hypoxic conditions within the stroma induce the production of suppressive cytokines — particularly interleukin-10 (IL-10) and transforming growth factor-β (TGF-β) — that inhibit immune cell activation and promote immune tolerance [30]. Thus, the stroma not only blocks immune cell entry but actively contributes to a tumor-permissive immunological landscape.

In addition to the physical barrier, PDAC is shaped by active immune suppression involving both innate and adaptive immune cells (Fig. 1). MDSCs are recruited into the tumor microenvironment by tumor-secreted growth factors such as granulocyte-macrophage colony-stimulating factor (GM-CSF). Once present, MDSCs inhibit T cell activation by depleting key amino acids (e.g., cysteine), generating reactive oxygen species (ROS), and secreting immunosuppressive cytokines including IL-4 and IL-10 [31, 32]. TAMs, another dominant myeloid population, are polarized toward a tumor-promoting M2 phenotype under the influence of cytokines such as IL-4, IL-13, and colony-stimulating factor 1 (CSF-1) [33]. These M2-like TAMs secrete TGF-β, vascular endothelial growth factor (VEGF), platelet-derived growth factor (PDGF) and MMPs, which together suppress T cell activity, support angiogenesis, and remodel tissue to facilitate tumor invasion [33, 34].

Dendritic cells (DCs), which are essential for antigen presentation and T cell priming, are markedly deficient in PDAC. Conventional DCs (cDCs) are not only scarce but often rendered dysfunctional by the local cytokine milieu. As a result, the priming of naive T cells and the initiation of effective adaptive immune responses are severely compromised. Enhancing DC recruitment and restoring their antigen-presenting capacity represent key strategies to reinvigorate immune surveillance in PDAC [35].

Adaptive immune cells are likewise impaired. NK cells, which mediate antigen-independent cytotoxicity, are poorly represented in PDAC and exhibit functional suppression due to elevated levels of TGF-β, IL-10, and indoleamine 2,3-dioxygenase (IDO) in the tumor microenvironment [36] (Fig. 1). Even when present, NK cells in PDAC show reduced expression of activating receptors and impaired cytokine production, limiting their tumor-killing capacity. CD8⁺ T cells—central to antitumor immunity—often fail to accumulate within tumor nests and are functionally exhausted. Meanwhile, Tregs accumulate in large numbers and suppress effector T cell responses by secreting TGF-β and IL-10, consuming interleukin-2 (IL-2), and inhibiting dendritic cell function via CTLA-4, is one of immune checkpoint binding CD80/CD86 on APC to inhibit antigen presenting [37, 38]. Additional immunosuppressive populations, such as Th17, Th22 cells, and regulatory B cells (Bregs), further reinforce this suppressive environment through the secretion of IL-17, IL-22, and IL-35 [39, 40].

In summary, myCAFs contribute to the formation of a dense extracellular matrix (ECM), which promotes abnormal angiogenesis and elevates interstitial pressure, thereby limiting immune cell infiltration. Concurrently, iCAFs secrete CXCL12 to hinder CD8⁺ T cell trafficking and recruit immunosuppressive cells such as MDSCs and Tregs, while also increasing levels of immunosuppressive cytokines like TGF-β and IL-10. TAMs further exacerbate the stromal barrier by releasing TGF-β and VEGF, enhancing both immune suppression and stromal remodeling. Collectively, these structural and immunological barriers exclude or impair the function of key immune effector cells, including CTLs, cDCs, and NK cells. As a result, conventional immunotherapies have demonstrated limited success in PDAC. Clinical trials using checkpoint inhibitors, such as anti–CTLA-4 and anti–PD-1/PD-L1 antibodies, have failed to produce meaningful response rates or survival benefits. For example, a Phase II trial of ipilimumab in metastatic PDAC demonstrated no objective responses, and trials with durvalumab, alone or in combination with tremelimumab, similarly showed minimal clinical impact [29, 41]. Other immunotherapies, including adoptive T cell therapies and cytokine treatments, have faced comparable challenges, often complicated by immune-related adverse events such as systemic inflammation or autoimmunity [42].

To overcome these limitations, next-generation immunotherapeutic strategies must address the unique features of the PDAC microenvironment. Nanotechnology provides a versatile and promising platform to do so. NPs can improve the pharmacokinetics and tumor-specific accumulation of immunomodulatory agents, reduce systemic toxicity, and facilitate targeted delivery to specific cell types or stromal compartments [43, 44]. Through size optimization, surface modification, and stimuli-responsive release mechanisms, NPs can penetrate fibrotic stroma, deliver therapeutic payloads to immunologically “cold” regions of the tumor, and co-deliver multiple synergistic agents—such as antigens, adjuvants, cytokines, or checkpoint inhibitors—within a single formulation [45, 46]. In the following sections, we explore how nanotechnology is being applied to reprogram the tumor microenvironment, enhance antigen presentation, stimulate effector immune cells, and unlock the full potential of immunotherapy in PDAC.

## Nanoparticle-enhanced immunotherapy for pancreatic cancer

Nanomaterials, defined by their nanoscale dimensions, possess unique physical and chemical properties that can be utilized for medical applications. NPs can be precisely engineered for disease-specific targeting and drug delivery, offering several therapeutic advantages: enhanced biocompatibility, reduced systemic toxicity, prolonged circulation time, and improved drug accumulation and cellular uptake at disease sites [43, 47]. These properties have been successfully exploited in NP-based cancer therapies, as evidenced by FDA-approved nanodrugs such as Doxil^®^ (liposomal doxorubicin) and Onivyde^®^ (liposomal irinotecan), which show superior safety profiles compared to conventional formulations [48, 49].

NPs are broadly classified into inorganic and organic types, each with distinct benefits and challenges. Inorganic NPs (e.g., carbon nanotubes, gold NPs, magnetic iron oxide, mesoporous silica) are valued in imaging and therapy for their optical, electrical, and magnetic properties. For example, superparamagnetic iron oxide NPs (SPIONs) are FDA-approved MRI (magnetic resonance imaging )contrast agents that enhance tumor imaging [50]. Inorganic NPs are also explored in photothermal therapy and as radiosensitizers [51, 52]. However, concerns about long-term biocompatibility, potential toxicity, and biodegradability can limit their clinical use.

In contrast, organic NPs (lipid-based or polymer-based) are composed of biocompatible, biodegradable materials and generally exhibit lower toxicity. Liposomes and polymeric NPs are especially notable for their ability to encapsulate both hydrophilic and hydrophobic drugs, as well as for their surface modifiability to enable targeted delivery. Liposomal formulations, with their cell membrane-like phospholipid bilayers, have achieved clinical success in cancer therapy [53, 54]. Similarly, polymer-based systems—particularly polymersomes or lipid–polymer hybrid NPs—can offer greater structural stability and reduced drug leakage compared to conventional liposomes, thus enhancing therapeutic efficacy in certain contexts [55–58]. Furthermore, hybrid NPs that combine lipid and polymer components are being developed to leverage the strengths of both materials and overcome individual limitations [55–57]. These advances in nanotechnology, especially when coupled with immunotherapeutic agents, hold strong potential for improving treatment outcomes in hard-to-treat cancers such as PDAC.

In the context of PDAC, immunotherapy faces substantial hurdles due to the hostile tumor microenvironment. NPs present a novel way to overcome these barriers by targeted delivery of immune-modulating agents into the tumor, thereby enhancing local immune activation while minimizing systemic side effects [59]. By using NPs to reprogram the tumor microenvironment or protect therapeutic cargo (like cytokines, antibodies, or nucleic acids) until they reach tumor sites, the efficacy of immunotherapies can be significantly improved. The integration of nanotechnology with immunotherapy is thus emerging as a powerful strategy against PDAC, utilizing unique capabilities of NPs to overcome drug delivery obstacles and immune evasion mechanisms.

Current nanotechnology-enhanced immunotherapy strategies for PDAC include: development of potent cancer vaccines, modulation of the immunosuppressive tumor microenvironment, direct stimulation of immune effector cells in the tumor, and targeting the desmoplastic stroma to improve treatment penetration, and we collected several nanotechnology-enhanced immunotherapy academic research in Table 2 (Table 2). Together, these approaches aim to prevent tumor immune escape and activate robust anti-tumor immune responses within the challenging PDAC microenvironment, offering new hope for improved patient outcomes.

**Table 2:** Preclinical NP-based immunotherapies in PDAC

Strategy	Therapeutic cargo	Nanomedicine	Administration route	Combination	Advantage	Limitation	Ref
**Cancer Vaccine Enhancement**							
**mRNA nanovaccine**	STING agonist cGAMP and KRAS or neoantigen-encoding mRNA	LNPs	Intravenous	Single	Protecting mRNA from degradation and improving dendritic cell uptake; enabling effective in situ tumor antigen expression and strong CTL activation​; inducing robust CD8 and CD4 T-cell responses in PDAC	LNPs often accumulate in liver and spleen, limiting lymph node and tumor delivery​; dense PDAC stroma and immunosuppressive cells impede T-cell infiltration even after vaccination​; risk of off-target innate immune activation; personalized vaccine manufacturing challenges​	[62] [65]
**“All-in-one” nanovaccine**	TLR4 agonist (MPLA) and Ψ/5meC-modified mRNA	LNPs	Intravenous	Single	Simultaneously delivering tumor antigens plus immune stimulants to address multiple immunosuppressive mechanisms​; Broadening the antigenic repertoire and shifts the tumor microenvironment toward a proinflammatory Th1-polarized state; enhancing T-cell immunogenicity​	Complex multi-component design requiring synchronized payload release​; co-delivered agents risk increased toxicity; manufacturing multi-component nanomedicines is technically challenging​	[64]
**Targeted peptide vaccine**	WT1 antigen	Ganglioside-functionalized liposomal NPs	N/A (in vitro)	N/A (in vitro)	Targeting vaccine to specialized APC subsets in lymphoid tissues; improving antigen presentation and T-cell priming​; enhancing tumor-specific CD8 T-cell responses and IFN-γ production; overcoming poor peptide immunogenicity	Efficacy limited to tumors expressing the target antigen and patients with appropriate HLA types; single-epitope approach risks narrow coverage of tumor heterogeneity; immune tolerance mechanisms in PDAC can dampen responses.	[76]
**Whole-cell lysate nanovaccine**	Tumor cell lysate and mtDNA	Cationic polymeric NPs	Intravenous	Single	Providing a broad spectrum of tumor antigens along with an intrinsic immunostimulatory danger signal (mtDNA) to promote antigen presentation​; enhancing dendritic cell uptake and maturation; inducing robust and durable tumor-specific T-cell responses	Complex antigen mixtures can complicate reproducibility and regulatory approval; immune tolerance mechanisms in PDAC can dampen responses.	[78]
**Immunosuppressive tumor microenvironment Modulation**							
**Induction of ICD**	Photosensitizer (PPa) + BRD4 inhibitor (JQ1)	HA-polymeric NPs	Intravenous	Single	Generating ROS for ICD; promoting DC maturation and CD8⁺ T-cell activation; reprograming tumor microenvironment	Dependent on NIR light; limited tissue penetration; risk of phototoxicity.	[88]
**Modulation of immunosuppressive pathways**	Activated oxaliplatin (1,2-diamminocyclohexane platinum(ii))	Mesoporous silica NPs	Intravenous	PD-1 antibody	Inducing ICD by releasing DAMPs; promoting DC maturation and CD8⁺ T-cell activation; reducing systemic toxicity through localized intratumoral delivery.	Limited efficacy as a monotherapy; risk of off-target effects on normal fibroblasts potentially promoting metastasis; intratumoral administration may not be feasible in clinical PDAC treatment.	[77]
	Docetaxel and IDO1 inhibitor NLG919	pH-responsive polyphenol-based NPs	Intravenous	Single	Remodeling the immunosuppressive tumor microenvironment by combining ICD-inducing chemotherapy with immune checkpoint inhibition; enhancing activation of DCs, macrophages, and CD8⁺ T cells.	Challenges in dual-drug encapsulation and formulation stability; potential systemic toxicities from combination therapy.	[90]
	TGF-β inhibitor (LY2157299) and PD-L1 siRNA	pH-responsive polymeric NPs	Intravenous	Single	Simultaneous targeting stromal and tumor immunosuppressive pathways; increasing T-cell infiltration and activation in tumor microenvironment	Redundant suppressive pathways may limit efficacy; complex dual payload delivery; other suppressive mechanisms may persist; limited distribution in fibrotic tumors.	[91]
	Oncolytic LTX-315 peptide and TGF-β1 siRNA	cRGD-Modified Hybrid NPs	Intravenous	Single	Specifically targeting the tumor microenvironment via αvβ3 integrin; suppressing angiogenesis; enhancing infiltration and activation of CD8⁺ T cells and NK cells; reprograming immunosuppressive stroma.	NP delivery efficiency may decrease following vascular normalization; mature vasculature may hinder extravasation; complex multi-cargo formulation; potential off-target effects of the oncolytic peptide on normal cells.	[92]
**Immune Effector Cell Stimulation**							
**Cytokine-based therapy**	IL-12 plasmid	Oncolytic virus vectors	Intravenous	Single	High local IL-12 expression with reduced systemic toxicity; promoting CD8 T cell and M1 macrophage activation.	Tumor transduction heterogeneity; viral vectors may be immunogenic; multiple doses may be required; non-specific innate immunity activation by plasmid.	[97]
	IL-12 plasmid	PQDEA polyplex NPs	Intravenous	Single	High local IL-12 expression with reduced systemic toxicity; promoting CD8 T cell and M1 macrophage activation.	Potential systemic toxicity; tumor transduction heterogeneity; viral vectors may be immunogenic; multiple doses may be required; non-specific innate immunity activation by plasmid.	[98]
	IL-2 and ICD inducers Pt-NHC	Multifunctional nanogels	Intravenous	Losartan	Sustained release of IL-2 and ICD inducers; enhancing lymphocyte activity and TAM repolarization.	Requires precise dosing to avoid toxicity; IL-2 may expand Tregs; uneven NP distribution in dense stroma.	[99]
	IL-12 and IL-27 mRNA	LNPs	Intratumoral	Single	Strong NK and CD8 T cell response with dual immune activator; reduced systemic toxicity by intratumoral injection	Local administration is impractical for treating PDAC.; tumor transduction heterogeneity	[105]
**NP-augmented checkpoint blockade**	Irinotecan	Liposomal irinotecan (Onivyde^®^)	Intravenous	CXCR4 inhibitor BL-8040; PD-1 antibody	Enhancing T-cell infiltration and activation, simultaneously targeting stromal and tumor immunosuppressive pathways;	Complex regimen; possible additive toxicities; other suppressive mechanisms may persist; limited distribution in fibrotic tumors.	[109]
	PD-L1 inhibitor miR-142, TLR7/8 agonist R848 and VCP/p97 inhibitor CB-5083	Solid LNPs	Intravenous	Single	Simultaneous suppressing PD-L1 and stimulating innate immunity; enhancing CD8 T-cell infiltration and activation	Complex regimen; limited benefit from single checkpoint targeting; uneven NP uptake may leave residual PD-L1 expression.	[110]
**TAM reprogramming**	CSF-1R -siRNA or PI3Kγ inhibitors NVP-BEZ 235	M2pep-decorated NPs	Intravenous	Single	Targeting M2-like macrophages for depletion or reprogramming; relieving immunosuppression and supporting T-cell based therapies.	Macrophage heterogeneity complicates targeting; risk of off-target effects and incomplete reprogramming; limited distribution in fibrotic tumors.	[117]
**Delivery of innate immune agonists**	STING agonists cdGMP and TLR4 agonists MPLA	LNPs	Intravenous	MEK Inhibitors trametinib; CDK4/6 Inhibitors palbociclib	Amplifying type I interferon signaling; activating DCs and macrophages; enhancing senescence-associated secretory phenotype (SASP) to boost CD8⁺ T cell responses.	Complex multi-agent regimen; risk of off-target effects on normal cells; limited delivery efficiency due to dense ECM.	[124]
	TLR7/8 agonist 3 M-052 and Irinotecan	Silicasomes	Intravenous	Single	Enhancing APC function through ICD signal induction; increasing CD8⁺/Treg cell ratio to promote antitumor immunity.	Potential off-target effects on normal tissues; limited tumor penetration in fibrotic microenvironments.	[125]
**Desmoplastic Stroma and Abnormal Vasculature Modulation**							
**Fibroblast-targeted therapy**	PD-1/PD-L1 inhibitor BMS-202 and IR-780	CAF-targeted photothermal liposome	Intravenous	Single	Targeting FAP- α fibroblast for specific delivery; decreased normal cell toxicities with photothermal therapy; enhancing CD8 T cell response	CAF heterogeneity; the risk of off-target effects on normal fibroblasts; limited feasibility of photothermal therapy in PDAC.	[127]
	IDO inhibitor 1-methyl-tryptophan and celastrol	HA-coated albumin NPs delivering	Intravenous	Single	Targeting CD44 + CAFs to reduce fibrosis and IDO-mediated immunosuppression; enhancing T-cell infiltration.	CAF heterogeneity; limited NPs distribution in dense stroma; risk of off-target uptake.	[128]
**TGF-β inhibition in fibrotic stroma**	α-mangostin or triptolide	CREKA-functionalized PEG-PLA NPs	Intravenous	Gemcitabine	Accumulating in fibrotic regions via fibrin–fibronectin binding; inhibiting CAF activation and enhancing chemo delivery.	Single-pathway inhibition may be insufficient; heterogeneous NPs penetration.	[129]
**ECM degradation via enzyme delivery**	Oncolytic vaccinia virus encoding the hyaluronidase (OVV-Hyal1)	Soluble hyaluronidase (Hyal1) enzyme gene​	Intratumoral	Doxorubicin; Gemcitabine; Liraglutide (GLP-1 analog); anti–PD-1; anti–CD47; CAR-T cells	Digesting hyaluronic acid (HA) to decompress vessels; enhancing perfusion and facilitating drug delivery and immune cell infiltration.	Risk of excessive stroma loss promoting invasion; enzyme must be precisely localized to tumor site; mechanism by which HA degradation boosts immune cell activity is not yet clarified	[132]
**RNAi Nanomedicines for Stromal Remodeling**	anti–miR-210 and KRAS^G12D^ siRNA	Cholesterol-modified CXCR4-targeting NPs	Intraperitoneal	Single	Targeting chemokine and hypoxia-associated pathways; reprograming PSCs and suppressing tumor proliferation; enhancing T cell infiltration and activation in the tumor microenvironment.	Redundant suppressive pathways may limit efficacy; complex dual payload delivery; other suppressive mechanisms may persist; limited distribution in fibrotic tumors.	[133]
	Hedgehog inhibitor LDE225 and SOX9 siRNA	Tumor membrane-coated NPs	Intravenous	Single	Suppressing MDSC infiltration; reducing fibrotic stroma; enhancing CD8⁺ T cell infiltration and immune activation.	Risk of off-target uptake; complex dual-drug encapsulation; restricted distribution in dense stroma.	[134]
**Vessel normalization + immune activation**	α-mangostin phosphate and LIGHT cytokine	Nano-sapper NPs	Intravenous	single	Improving vascular integrity and oxygenation while recruiting immune cells; enhancing perfusion and T-cell infiltration	Normalization window is short; NP delivery may decline post-normalization; mature vessels may limit NP extravasation; precise dosing required.	[140]
**Vessel normalization + chemotherapy**	Cyclopamine and paclitaxel (PTX)	Polymeric micelle	Intravenous	PD-1 antibody	Inhibiting stromal and angiogenic pathways; enhancing perfusion and T-cell infiltration; sensitizing tumors to ICI.	Normalization window is short; NP delivery may decline post-normalization; mature vessels may limit NP extravasation; precise dosing required.	[142]

### Nanotechnology for enhancing cancer vaccine efficacy in PDAC

Cancer vaccines, a critical arm of immunotherapy, aim to train the immune system to recognize and destroy cancer cells by presenting tumor-associated antigens (TAAs) or tumor-specific antigens (TSAs) to immune cells. However, traditional cancer vaccine platforms (whether peptide, protein, or nucleic acid based) often face limited efficacy, especially in the immunosuppressive tumor microenvironment of PDAC, where immune activation is blunted [15, 60]. Nanotechnology, with its ability to improve antigen delivery, stability, and immune cell targeting, has emerged as a game-changer to overcome these hurdles and potentiate vaccine responses [61].

#### Nucleic acid–based cancer vaccines

**Fig. 2:**
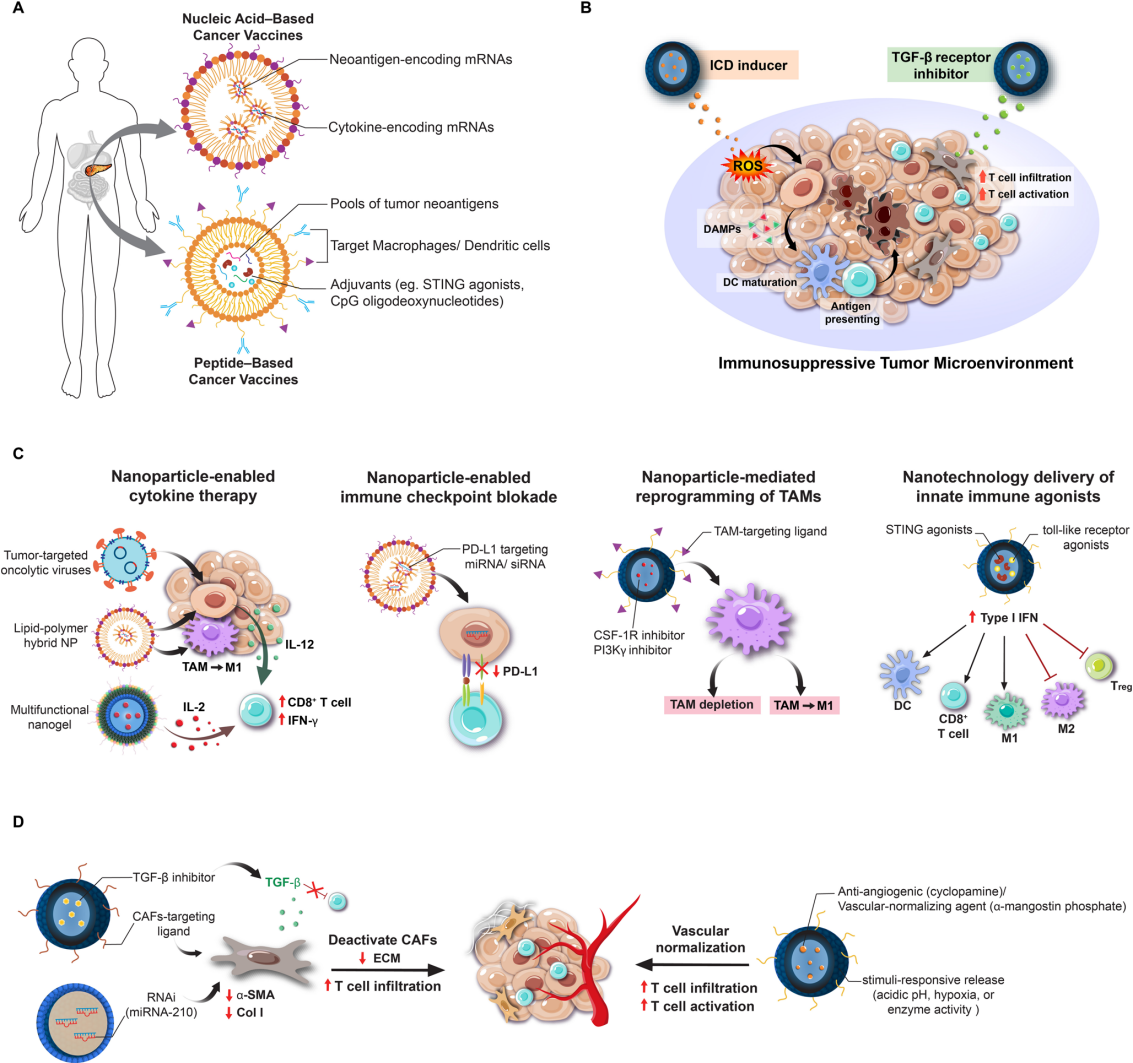
Nanotechnology-enhanced immunotherapy strategies for PDAC. **(A)** NP-enabled cancer vaccines enhance antigen delivery and immune activation in PDAC. Lipid and polymeric NPs protect nucleic acid- and peptide-based vaccines from degradation, improve lymphoid tissue targeting, and potentiate dendritic cell (DC) priming of cytotoxic T cells. **(B)** Remodeling the immunosuppressive tumor microenvironment using NPs promotes antigen release, reduces stromal barriers, and enhances infiltration of antitumor immune cells. Strategies include induction of immunogenic cell death (ICD), blockade of immunosuppressive signaling pathways (e.g., TGF-β, PD-L1), and reprogramming of cancer-associated fibroblasts (CAFs). **(C)** NP-facilitated immune effector cell stimulation enhances T and NK cell responses in PDAC. Controlled delivery of cytokines (e.g., IL-2, IL-12) and checkpoint inhibitors via NPs reduces systemic toxicity and improves therapeutic efficacy. **(D)** Nanotechnology-based modulation of the desmoplastic stroma and aberrant vasculature alleviates physical barriers, normalizes perfusion, and facilitates NP and immune cell infiltration into tumors, thus enhancing the efficacy of combination immunotherapies

NP platforms offer significant advantages for delivering nucleic acid vaccines—such as mRNA or DNA—in the context of PDAC, where the immunosuppressive tumor microenvironment and rapid nucleic acid degradation severely limit vaccine efficacy (Fig. 2A). Encapsulation of mRNA in lipid-based NPs protects the cargo from enzymatic degradation in circulation and facilitates efficient uptake by antigen-presenting cells (APCs), particularly DCs. A notable example is Moderna’s mRNA-5671, a lipid NP-formulated mRNA vaccine targeting four common KRAS mutations (G12D, G12V, G13D, and G12C)—driver mutations found in lung, colorectal, and pancreatic cancers [62]. The lipid carrier stabilizes the mRNA and promotes intracellular delivery, enabling in situ antigen production and effective priming of cytotoxic T lymphocytes (CTLs) against KRAS-mutant tumor cells. Building upon this concept, a recent study by Rojas et al. demonstrated the use of a lipoplex NP vaccine encoding patient-specific neoantigen mRNAs for PDAC. The lipoplex NP cancer vaccine, when co-delivered with atezolizumab (an anti–PD-L1 antibody) and mFOLFIRINOX chemotherapy, induced robust CD8⁺ and CD4⁺ T cell responses within the tumor microenvironment. These results highlight the potential of nanotechnology-based strategies to synergize effectively with immune checkpoint blockade in PDAC [63].

In addition to enhancing mRNA stability and intracellular delivery, next-generation “all-in-one” nanovaccine platforms are designed to address multiple immunological challenges simultaneously by co-delivering tumor antigens and immunomodulatory agents—such as Toll-like receptor (TLR) agonists, cytokine-encoding mRNAs, or ICIs—within a single NP. This integrated approach is particularly relevant for PDAC, where both poor T cell priming and strong immunosuppression coexist [64]. By packaging multiple neoantigens alongside adjuvants or inhibitory pathway modulators, these multifunctional NPs broaden the antigenic repertoire, enhance immunogenicity, and shift the tumor microenvironment toward a more proinflammatory, T helper 1–dominant state. Recent preclinical studies have also demonstrated that such dual-function NPs, for example those encoding both tumor antigens and PD-L1–silencing RNA or IL-12 mRNA, can augment effector T cell responses while mitigating immune exhaustion [65].

Despite recent advances, several challenges limit the efficacy of NP-based nucleic acid vaccines in PDAC. Systemically administered lipid NPs often accumulate in the liver and spleen, reducing delivery to tumor-draining lymph nodes or intratumoral APCs [66]. The dense stroma and abnormal vasculature of PDAC further hinder intratumoral penetration and lymphatic drainage, while the immunosuppressive tumor microenvironment—rich in Tregs, MDSCs, and M2 macrophages—can inactivate cytotoxic T cells even after successful priming [67]. Additionally, lipid NPs and unmethylated RNA can trigger off-target innate immune activation, leading to systemic inflammation [68]. Tumor antigen selection remains a critical hurdle, as heterogeneity of PDAC and diverse human leukocyte antigen (HLA) profiles complicate neoantigen targeting. Repeated dosing may trigger anti-vector immunity or accelerated blood clearance, particularly in PEGylated formulations [69]. Finally, the individualized nature of personalized mRNA vaccines poses significant manufacturing, regulatory, and scalability challenges.

Future developments aim to further optimize NP vaccine delivery and potency for PDAC. One direction is to enhance lymph node targeting– for instance, engineering NPs with optimized size (~ 10–50 nm), surface charge, and ligand functionalization (e.g., mannose, antibodies to DC-SIGN) to improve trafficking to lymphoid tissues and uptake by DCs [70, 71]. Personalization will remain key– advances in neoantigen prediction and sequencing can feed directly into custom NP vaccines produced rapidly for post-surgery PDAC patients​. To address tumor access issues, researchers are exploring direct administration routes (such as intranodal or intratumoral injection of vaccine NPs) to bypass systemic barriers and fibroblastic stroma [72]. Improved nanocarrier designs, including biodegradable polymers or exosome-mimicking NPs, may offer better penetration into pancreatic tumors or enhanced biocompatibility [73]. In summary, future nano-vaccines for PDAC will likely be multi-component and personalized, integrating antigen delivery with smart strategies to overcome immunosuppressive tumor microenvironment, thereby enhancing the vaccine-primed T cells, leading to a sustained and effective antitumor response against this highly resistant malignancy.

#### Peptide-based cancer vaccines

Peptide-based vaccines, which introduce defined tumor antigen epitopes to activate T cell responses, represent an attractive cancer vaccine modality owing to their safety, specificity, and ease of synthesis. However, their clinical utility—particularly in immunologically “cold” tumors like PDAC—has been constrained by inherently poor immunogenicity. In such contexts, peptide vaccines often fail to elicit robust immune activation. The low immunogenicity of peptide antigens, coupled with the immunosuppressive tumor microenvironment of PDAC, poses a major barrier to effective peptide vaccine-induced anti-tumor immunity [74].

Nanotechnology-based strategies offer promising solutions to overcome the immunosuppressive barriers of PDAC by improving antigen delivery, enhancing uptake by APCs, and providing immunostimulatory signals (Fig. 2A). One compelling example involves targeting the Wilms Tumor 1 (WT1) antigen—a transcription factor aberrantly overexpressed in the majority of PDAC tumors but largely absent in normal pancreatic tissue. WT1 is not only associated with tumor aggressiveness and poor prognosis but is also highly immunogenic, capable of eliciting potent CD8⁺ T cell responses [75]. Recognized by the National Cancer Institute as a high-priority tumor-associated antigen, WT1 has been the focus of multiple peptide-based vaccine trials, which have demonstrated safety and immunological activity in PDAC patients, including delayed disease progression in responders. To further enhance WT1 vaccine efficacy, researchers have developed ganglioside-functionalized liposomes encapsulating WT1-derived peptides. These NPs are engineered to target CD169⁺ macrophages and Axl⁺ DCs—specialized APC subsets enriched in lymphoid tissues—thereby improving antigen presentation and T cell priming. In preclinical models, this targeted delivery system significantly enhanced WT1-specific CD8⁺ T cell responses and IFN-γ production, overcoming the inherent limitations of peptide immunogenicity [76]. Collectively, these findings underscore WT1 as a clinically relevant and immunologically actionable antigen in PDAC and highlight how nanotechnology can potentiate peptide-based cancer vaccines by improving delivery, presentation, and immune activation.

In addition to tumor antigens like WT1, fibroblast activation protein-α (FAP), a marker of CAFs, has recently emerged as a novel immunotherapeutic target in desmoplastic tumors. Shin et al. developed a lipid nanovaccine displaying immunodominant FAP-specific CD8⁺ T cell epitopes on the surface, which, upon immunization, selectively depleted FAP⁺ CAFs, reduced ECM deposition, and enhanced T cell infiltration within the tumor microenvironment​ [77]. In murine models, this FAP-targeted nanovaccine not only slowed tumor growth and metastasis but also synergized with chemotherapy by improving drug penetration through stromal remodeling​. These findings support CAF-targeted peptide nanovaccines as a complementary strategy to potentiate immunogenicity and improve therapeutic outcomes in PDAC.

Beyond single-epitope vaccines, nanotechnology also enables the co-delivery of complex antigenic and immunostimulatory payloads to further broaden and potentiate anti-tumor immunity. For instance, Shang et al. developed cationic poly(lactic-co-glycolic acid) (PLGA) NPs co-loaded with whole tumor cell lysates and mitochondrial DNA (mtDNA), generating a neoantigen-rich, immunogenic vaccine formulation for PDAC [78]. This nanovaccine significantly enhanced dendritic cell uptake and maturation, driving robust antigen presentation and potent activation of tumor-specific T cells. Compared to administration of vaccine components alone, the NP-based system elicited more durable and effective T cell–mediated immune responses, translating into superior therapeutic efficacy in preclinical PDAC models. These findings underscore the value of nanotechnology in overcoming immune tolerance and suppression within the PDAC tumor microenvironment, enabling more sustained and systemic anti-tumor immunity from cancer vaccines.

Despite recent progress, peptide nanovaccines for PDAC still face significant challenges. Suitable tumor antigens are limited and often patient-specific. Shared targets like WT1 or mesothelin may benefit only a subset of patients with high antigen expression and compatible HLA types. This necessitates either personalized epitope selection or multi-peptide formulations, which complicate dosing, stability, and regulatory pathways. While NPs can co-deliver multiple peptides, overloading may dilute efficacy or destabilize the formulation [79]. Immunologically, peptides delivered without sufficient co-stimulation—particularly in TGF-β and IL-10–rich tumor microenvironment of PDAC—may induce tolerance or expanding Tregs [80]. Moreover, robust CD8⁺ T-cell activation depends on efficient endosomal escape of NP-delivered antigens within DCs to enable cytosolic processing and MHC class I cross-presentation. Without this, antigens are degraded in lysosomes, severely limiting antigen presentation and anti-tumor T cell responses [81]. NPs may also be sequestered by stromal macrophages or non-target endothelial cells before reaching DCs, further reducing vaccine efficiency [82]. Safety is another concern, as peptides with homology to self-antigens may trigger autoimmunity, indicating the need for precise tumor-specific epitope selection. In summary, while nanocarriers improve peptide vaccine delivery and immunogenicity, they do not fully overcome the limitations posed by antigen heterogeneity, immune suppression, and interpatient variability in PDAC.

To enhance the efficacy of peptide-based nanovaccines in PDAC, future strategies are focusing on multi-epitope designs and combination therapies. Instead of single peptides, NPs are being formulated with pools of shared and personalized neoantigens to induce broad, polyclonal T cell responses and reduce immune escape. These vaccines are often combined with potent adjuvants—such as STING (Stimulator of Interferon Genes) agonists or CpG oligodeoxynucleotides—to activate DCs and enhance T cell priming [83]. Biomaterial-based depots, including injectable scaffolds or hydrogels, are also being developed to enable sustained NP vaccine release and continuous immune stimulation, which may be particularly beneficial in overcoming immune tolerance of PDAC [84]. Advances in neoantigen prediction and rapid peptide synthesis will facilitate personalized vaccine production during perioperative or adjuvant therapy windows. Combining peptide nanovaccines with agents like ICIs or stroma-modulating drugs (e.g., anti-PD-1 or CXCR4 inhibitors) is expected to further boost immune infiltration and tumor control. Additionally, virus-like NP platforms are being explored to enhance immunogenicity through their inherent structural mimicry of pathogens [85]. Together, these innovations aim to generate robust and durable T cell responses against PDAC.

In summary, NPs have emerged as powerful tools to stabilize vaccine antigens, promote lymphoid tissue targeting, and boost immune activation where traditional vaccines fall short. Lipid NPs for mRNA delivery and polymeric or liposomal carriers for peptide vaccines exemplify the transformative potential of nanotechnology to improve cancer vaccine outcomes. By enabling targeted delivery, protecting antigens from degradation, and co-delivering adjuvants to stimulate APCs, these approaches significantly enhance immune recognition of PDAC. Nanovaccines and related technologies thus hold promise to significantly improve therapeutic outcomes in PDAC and other traditionally “cold” tumors that have not responded to conventional immunization strategies.

### Nanotechnology for modulating the immunosuppressive tumor microenvironment

The immunosuppressive tumor microenvironment of PDAC represents a major obstacle to effective immunotherapy. Hallmarked by dense fibrotic stroma, immune-excluding physical barriers, and immunoregulatory signaling networks, the PDAC tumor microenvironment actively suppresses T cell activation, infiltration, and effector function [86]. To overcome these barriers, emerging strategies focus on reprogramming the tumor microenvironment to foster an immunostimulatory milieu capable of supporting robust anti-tumor immune responses. Nanotechnology offers a versatile platform to precisely deliver therapeutic agents that can reshape the immune landscape of PDAC, minimizing off-target effects and enhancing treatment efficacy.

One promising approach involves the induction of immunogenic cell death (ICD)—a form of tumor cell demise that actively stimulates the immune system (Fig. 2B). Unlike conventional cytotoxicity, ICD leads to the release of TAAs and damage-associated molecular patterns (DAMPs), such as calreticulin, ATP, and HMGB1. These signals activate pattern recognition receptors (e.g., TLRs, nucleotide-binding oligomerization domain-like receptors (NOD-like receptor)) on APCs, driving dendritic cell maturation and effective T cell priming [87]. ICD inducers are broadly categorized into Type I (inducing moderate endoplasmic reticulum stress (ERS)) and Type II (triggering severe ERS), with the latter generally eliciting stronger immunostimulatory effects.

NPs can enhance ICD induction by selectively delivering ICD-inducing agents to tumor sites, maximizing tumor immunogenicity while reducing systemic toxicity. For instance, Sun et al. developed hyaluronic acid (HA)-based polymeric NPs co-encapsulating a photosensitizer (pyropheophorbide a, PPa) and a BRD4 inhibitor (JQ1) for combinatorial photoimmunotherapy in PDAC. Upon near-infrared light irradiation, PPa generated intracellular ROS, promoting oxidative damage and robust ICD in tumor cells. Simultaneously, JQ1 suppressed oncogenic pathways (such as c-Myc) and downregulated immune checkpoint molecules like PD-L1. This dual-functional nanoplatform not only triggered immunogenic tumor cell death but also alleviated key immunosuppressive mechanisms, leading to enhanced CD8⁺ T cell activation, reduced metastasis, and prolonged survival in PDAC-bearing mice [88].

Chemotherapy-induced ICD has emerged as a key mechanism by which nanomedicine can convert immune-inert tumors into immunogenic ones. A platinum-loaded mesoporous silica “silicasome” was shown to induce robust ICD in PDAC, as evidenced by cell-surface calreticulin exposure and release of danger signals like ATP and HMGB1​​ [89]. This ICD response facilitated antigen presentation by DCs and primed a potent CD8⁺ T cell attack, effectively converting an immunologically “cold” tumor into a “hot” one​. In an orthotopic PDAC model, delivering the silicasome together with anti–PD-1 checkpoint blockade produced synergistic anti-tumor effects, resulting in greater tumor regression, reduced metastatic spread, and a significant survival benefit compared to chemotherapy alone​. Likewise, a polyphenol-based NP co-delivering docetaxel and an IDO1 inhibitor provoked ICD and relieved immunosuppression in PDAC, resulting in enhanced intratumoral CD8⁺ T cell infiltration and marked tumor growth inhibition in preclinical models​ [90].

Beyond inducing ICD, nanotechnology also enables the targeted modulation of immunosuppressive pathways that hinder immune cell infiltration and effector function within tumors. Even in the presence of circulating tumor-specific T cells, their entry into the tumor microenvironment is often obstructed by dense stroma and inhibitory cytokines. To overcome these barriers, NPs can be engineered to deliver agents that disrupt immunosuppressive signaling. For instance, a pH-responsive polymeric NP system was designed to co-deliver a TGF-β receptor inhibitor (LY2157299) and PD-L1-targeting siRNA into pancreatic tumors (Fig. 2B). The TGF-β inhibitor reduced fibroblast-mediated immunosuppression, while the siRNA suppressed PD-L1 expression on tumor cells, synergistically enhancing intratumoral T cell infiltration and cytotoxicity [91]. In another study, Phung et al. developed polymer–lipid hybrid NPs composed of PLGA, DSPE-mPEG, and cRGD–DSPE-PEG for targeted delivery of the oncolytic peptide LTX-315 and TGF-β1 siRNA (LTX/siR-NPs). The cRGD motif directed NPs to αvβ3 integrin, overexpressed on angiogenic vasculature, exerting antiangiogenic effects. Silencing TGF-β1 enhanced CD8⁺ T cell and NK cell infiltration and activation, reprogrammed the immunosuppressive microenvironment, and suppressed tumor growth in PDAC when combined with oncolytic therapy [92].

Collectively, these studies illustrate the multifaceted potential of NPs to reprogram the PDAC tumor microenvironment. By inducing immunogenic tumor cell death, neutralizing suppressive cytokines and checkpoints, and facilitating effector immune cell infiltration, nanotechnology transforms the tumor microenvironment from an immune-excluded, suppressive environment into one that is more inflamed and immunologically accessible. Crucially, the targeted delivery afforded by NPs confines immune modulation to the tumor site, mitigating systemic immune activation and reducing immune-related adverse effects (IrAEs). Such tumor microenvironment remodeling lays the foundation for enhancing the efficacy of complementary immunotherapies—such as cancer vaccines, ICIs, and adoptive T cell therapies—bringing the goal of durable anti-tumor immunity in PDAC and reprograming an immune “cold” PDAC tumor into a more inflamed, immune-accessible state.

### Nanotechnology for stimulating immune cells within the PDAC tumor microenvironment

Nanotechnology also provides innovative means to directly stimulate anti-tumor immune cells in the PDAC microenvironment. Traditional immunotherapies such as systemic cytokine administration or checkpoint inhibitors often suffer from dose-limiting toxicities and poor tumor penetration in PDAC. By using NPs as delivery vehicles or functional agents, we can concentrate immune stimulators in the tumor and reduce off-target effects. NPs can carry cytokines, nucleic acids, or drugs that activate immune cells (T cells, NK cells, macrophages) or relieve immune inhibition, thereby converting the “cold” PDAC tumor microenvironment into an immunologically active “hot” state (Fig. 2C).

#### Nanoparticle-enabled cytokine therapy

Cytokines are powerful immunomodulators that can activate various arms of the immune system, particularly T cells and NK cells. However, their clinical application in cancer has been limited by rapid clearance from circulation and severe systemic toxicities at therapeutically effective doses. For example, IL-2 and IFN-α were among the first cytokines approved for cancer treatment in the 1980s, showing efficacy in melanoma, renal cell carcinoma, and certain leukemias [93, 94]. Despite their initial promise, only a few cytokine therapies have gained approval over the past decades, largely due to their narrow therapeutic windows and limited efficacy in solid tumors. Nanotechnology provides an opportunity to revisit cytokine therapy by enhancing delivery, stability, and tumor localization. One widely explored strategy is to encapsulate cytokines or their gene-encoding plasmids into NPs to enable sustained, tumor-targeted release and minimize systemic exposure.

IL-12 is a potent cytokine that can activate NK and CD8⁺ T cells and stimulate IFN-γ production. However, systemic IL-12 therapy is associated with life-threatening inflammatory toxicities, such as cytokine release syndrome [95, 96]. To address this, Wang et al. utilized tumor-targeted oncolytic viruses carrying IL-12 plasmid DNA, enabling selective IL-12 production within PDAC tumors. These viruses functioned as NP-like carriers that preferentially infected tumor cells, resulting in localized IL-12 expression, increased intratumoral CD8⁺ T cells and IFN-γ levels, and prolonged survival, all with reduced systemic toxicity [97]. Similarly, another study encapsulated IL-12 plasmids in a lipid–polymer hybrid NP (PQDEA polyplex), which could transfect both tumor cells and TAMs. This delivery system remodeled the tumor microenvironment by enhancing CD8⁺ T cell responses and promoting M1 polarization of TAMs, while reducing immunosuppressive MDSCs and Tregs. The combined effect led to significant tumor regression in PDAC models [98].

Building upon this concept, our group developed a multifunctional nanogel (IL2-Pt@Nanogel) designed for combinatorial chemoimmunotherapy. This platform co-delivers recombinant IL-2 and a platinum-based chemotherapeutic agent (Pt-NHC), which functions as a type II ICD inducer. Upon accumulation in the PDAC tumor microenvironment, the nanogel facilitates the sustained release of IL-2 to stimulate T and NK cell activation, while Pt-NHC induces ICD in tumor cells, leading to the release of DAMPs. This dual action not only enhanced lymphocyte-mediated cytotoxicity but also reprogrammed TAMs from the immunosuppressive M2 phenotype toward a pro-inflammatory M1 state. The combined effects resulted in robust tumor suppression and prolonged survival in murine PDAC models [99]. These works show how NP-based cytokine delivery can extend cytokine half-life, localize immune activation to the tumor site, and minimize systemic toxicity—ultimately highlighting the potential of nanoplatforms to transform cytokines into effective immunotherapeutic agents for PDAC.

Despite their promise, cytokine nanotherapies in PDAC face several challenges. Achieving optimal cytokine concentration and timing is critical—subtherapeutic doses are ineffective, while rapid release may trigger systemic toxicity, immune desensitization, or off-target effects. The dense PDAC stroma can further impede uniform NP distribution, resulting in uneven cytokine exposure and patchy immune activation. Moreover, the immunosuppressive tumor microenvironment may counteract cytokine effects; for example, IL-2 can expand effector T cells but also promotes Treg proliferation, particularly in TGF-β–rich environments [100]. To overcome this, IL-2–releasing NPs may need to be combined with Treg-blocking agents such as low-dose cyclophosphamide or anti-CD25 antibodies [101]. Repeated dosing may also trigger immunogenicity, with anti-cytokine or anti-NP antibodies potentially diminishing efficacy. Precision targeting remains difficult—different immune subsets require distinct cytokines (e.g., IL-15 for NK cells, IL-2 or IL-21 for T cells), and indiscriminate delivery may lead to unintended immune exhaustion or suppression. Finally, clinical translation requires overcoming manufacturing hurdles: producing GMP-compliant NPs with large protein payloads, ensuring cytokine stability, and achieving consistent release profiles across batches are essential for regulatory approval.

Future directions in cytokine delivery for PDAC focus on enhancing tumor-specific immune stimulation while minimizing systemic toxicity. One promising strategy involves activatable cytokine prodrugs, in which cytokines are fused to tumor-specific peptides and remain inactive until cleaved by tumor-associated proteases [102–104]. NPs can localize and concentrate these pro-cytokines within the tumor, reducing off-target effects. Another approach employs NPs to deliver cytokine-encoding DNA or mRNA, transforming transfected tumor cells into local cytokine-secreting factories. For example, intratumoral injection of mRNA-lipid NPs encoding IL-12 and IL-27 can establish a sustained pro-inflammatory niche, promoting T cell recruitment and activation [105]. Locoregional delivery platforms, such as hydrogel depots or catheter-based infusion into the pancreatic artery, further refine spatial control [106]. Combinatorial regimens such as pairing cytokine-releasing NPs with immunogenic chemotherapy, checkpoint blockade, or STING agonists has shown synergistic antitumor effects in preclinical PDAC model [107]. Advances in nanocarrier design—such as tumor-penetrating or stimuli-responsive NPs—are also improving intratumoral distribution and on-demand cytokine release. As understanding of the PDAC immune landscape deepens, novel cytokine targets will emerge, and nanotechnology will remain central to their precise delivery. Collectively, these innovations position NP-mediated cytokine therapy as a promising strategy to reprogram immune-inert PDAC and enhance the efficacy of immunotherapy.

#### Nanotechnology-enhanced immune checkpoint blockade

ICIs such as anti-PD-1, anti-CTLA-4, and anti-PD-L1 antibodies have revolutionized cancer therapy by unleashing T cells that were being “braked” by tumors. Multiple ICIs are FDA-approved for various cancers [108]. In PDAC, however, single-agent checkpoint blockade has largely failed to yield benefits, due to the lack of pre-existing T cell immunity and the suppressive nature of PDAC. Nanotechnology can assist checkpoint blockade in two main ways: combining ICIs with other therapies in nanocarriers, and delivering checkpoint-silencing agents directly to tumor cells or immune cells.

Combining ICIs with nanocarriers for other drugs can create synergistic regimens. For example, a clinical trial (NCT02826486) in metastatic PDAC tested a combination of the CXCR4 antagonist BL-8040 (which mobilizes immune cells into the tumor) with the anti-PD-1 antibody pembrolizumab and liposomal irinotecan (Onivyde^®^) chemotherapy​ [109]. The rationale was that BL-8040 in a nanocarrier would disrupt the chemokine gradients retaining T cells in stroma, Onivyde would debulk the tumor and induce antigen release, and pembrolizumab would prevent remaining tumor cells from inhibiting T cells. This chemo-immunotherapy nanocombination showed enhanced immune cell infiltration and was tolerated, pointing to improved efficacy when checkpoint blockade is paired with NP-mediated stromal modulation​.

Another approach is NP-mediated gene silencing of checkpoints. Lo et al. developed solid lipid NPs (SLNs) loaded with a microRNA (miR-142) targeting PD-L1, plus an immunostimulatory TLR7/8 agonist (R848) and a stress inducer (CB-5083) [110]. These SLNs were designed to enter tumor cells and macrophages, knock down PD-L1 expression, and simultaneously activate innate immune pathways. In PDAC models, treatment with the SLNs led to decreased PD-L1 on tumor cells, increased CD8⁺ T cell infiltration, and tumor regression. By delivering a PD-L1 inhibitor directly as a nucleic acid rather than an antibody, the NPs achieved checkpoint blockade from “within” the tumor, potentially overcoming poor antibody penetration.

Despite promising advances, several challenges remain in applying nanotechnology to checkpoint blockades in PDAC. PDAC frequently engages multiple immunosuppressive pathways; targeting a single checkpoint (e.g., PD-L1) may be insufficient. However, the complexity of co-delivering multiple agents within a single NP demands precise coordination of dosing and release kinetics to ensure therapeutic synergy. Additionally, combining ICIs with other potent agents risks cumulative immune related toxicity. Manufacturing multi-component nanomedicines is also technically challenging and subject to regulatory hurdles. NPs also face physical constraints—loading large proteins like full-length antibodies is difficult, often requiring alternative formats such as nanobodies or mRNA-encoded antibodies, whose clinical efficacy must still be validated. Gene-silencing strategies (e.g., siRNA or miRNA delivery) face barriers in achieving efficient, uniform uptake across the tumor, potentially leading to incomplete checkpoint inhibition. Importantly, patient selection remains critical—checkpoint blockade is unlikely to succeed in immune-desert tumors without concurrent strategies to induce T cell priming.

Future directions for nanotechnology-enhanced checkpoint immunotherapy in PDAC focus on refining delivery platforms and expanding target pathways. Biomimetic NPs, such as T cell membrane-coated vesicles bearing surface PD-1, may serve as decoys to bind tumor PD-L1 and achieve deep tumor penetration [111, 112]. Targeted NPs are also being designed to home in on PD-1⁺ T cells and co-deliver stimulatory agents (e.g., IL-15 or 4-1BB agonists), thereby coupling checkpoint relief with T cell reactivation [113]. Beyond PD-1/PD-L1, nanocarriers enable combinatorial targeting of non-traditional checkpoints (e.g., LAG-3, TIM-3), metabolic suppressors (e.g., IDO, arginase), or immunosuppressive cytokines like TGF-β. For instance, a single NP could co-deliver a TGF-β receptor inhibitor and an anti–PD-L1 nanobody to concurrently disrupt soluble and surface-mediated immunosuppression—an approach already showing synergy in preclinical PDAC models [114]. Integration with vaccines and cell therapies is another emerging strategy. NPs could transiently deliver immune modulators (e.g., RNA encoding costimulatory molecules or checkpoint silencers) to engineered CAR-T or TCR-T cells, enhancing their function within the tumor microenvironment [115, 116]. Overall, NP strategies can amplify ICI therapy by multi-pronged attacks: altering the tumor microenvironment to be more receptive to T cells, and directly interfering with checkpoint signals in the tumor. This is especially valuable for PDAC, where simply blocking PD-1/PD-L1 has proven insufficient without additional immune activation or tumor debulking.

#### Nanoparticle-mediated reprogramming of tumor-associated macrophages

TAMs in PDAC are abundant immune suppressors that help tumors evade anti-tumor immunity. These macrophages are usually polarized to an M2-like, tumor-promoting phenotype by signals in the PDAC microenvironment (e.g. IL-4, IL-13, CSF-1), leading them to secrete factors like IL-10 and TGF-β that suppress T cell activity and support fibrosis and angiogenesis. NPs provide a powerful platform to modulate TAMs in PDAC, either by depleting them or re-educating them toward an M1 (pro-inflammatory, anti-tumor) state. One inherent advantage is that NPs naturally tend to accumulate in macrophages– as phagocytic cells, TAMs readily engulf NPs, which can be exploited for targeted drug delivery: NPs can serve as trojan horses carrying agents that kill or reprogram TAMs upon ingestion​. This macrophage uptake can be enhanced by decorating NPs with ligands that bind M2 macrophage markers ​ [117, 118]. For example, an M2pep peptide that binds mannose receptor on M2 TAMs​. In PDAC models, such targeted NPs have homed to TAM-rich regions and delivered payloads like CSF-1R inhibitors (to block macrophage survival signals) and PI3Kγ inhibitors (to push macrophages toward M1 polarization), resulting in TAM depletion or phenotype switching in situ ​​ [117]. The immunological impact of this is significant: relieving macrophage-mediated suppression leads to higher T cell infiltration and activity.

Another advantage is synergy with other therapies. TAM modulation by itself can slow tumor growth (since M1 macrophages can directly attack tumor cells or present antigens), but it truly shines when combined with T-cell–targeted therapies. For instance, converting PDAC TAMs to an M1 state via NPs greatly improved the efficacy of adoptive T cell transfer and checkpoint blockade in preclinical studies. Thus, nanotechnology-enabled TAM reprogramming “softens the ground” for other immunotherapies. Moreover, some NP approaches arm the macrophages with new weapons: delivering TLRs agonists or STING agonists into TAMs can simulate them to produce and release type I interferons and other stimulatory signals, effectively turning the tide against the tumor. In summary, NP strategies that target TAMs can alleviate immune suppression, promote tumoricidal inflammation, and coordinate with T cell-based therapies– a multifaceted benefit especially relevant in macrophage-rich tumors like PDAC.

TAM-targeted nanotherapies face several challenges. First, macrophages in PDAC are heterogeneous, with some subsets exhibiting antitumor roles; indiscriminate depletion may disrupt beneficial functions such as debris clearance or immune coordination. Moreover, many TAM-targeting NPs are nonspecific and may be taken up by other phagocytes, raising the risk of off-target effects and systemic toxicity. Additionally, even when TAMs are successfully reprogrammed to a pro-inflammatory M1 phenotype, tumors may adapt by recruiting new monocytes or inducing reversion to M2 through IL-10 and CSF signals, necessitating repeated dosing. However, frequent administration may trigger anti-NP immunity and accelerate clearance. Efficient delivery of genetic payloads (e.g., miRNA, siRNA) into macrophages also remains technically demanding due to endosomal degradation. Clinically, monitoring TAM modulation is invasive and lacks standardized biomarkers. Finally, because PDAC relies on multiple immunosuppressive mechanisms, TAM reprogramming alone may be insufficient, requiring combination strategies that also address other suppressive cell types such as Tregs or MDSCs.

Future strategies for TAM modulation in PDAC focus on enhancing specificity and combining therapies. Efforts are underway to identify markers unique to immunosuppressive TAMs—such as folate receptor-β or scavenger receptors—for targeted NP delivery [119]. Rather than depleting all macrophages, reprogramming TAMs to a tumoricidal M1 phenotype using NP-delivered agents (e.g., CD40 agonists, PPARγ modulators, or IL-12 mRNA) is gaining traction [120]. Such approaches preserve macrophage-mediated functions while boosting local immune activation. Sequential treatment regimens—first reprogramming TAMs, then introducing cancer vaccines, T cell therapies, or checkpoint inhibitors—may enhance antitumor efficacy. NPs can be tailored for each step, making them ideal for combination strategies. Biomarker-guided approaches, such as PET imaging to identify high-TAM tumors, could enable personalized therapy [121]. Emerging cell-based vectors, such as drug-loaded monocytes that differentiate into TAMs at the tumor site, offer further targeting precision [122]. Finally, coupling TAM modulation with stroma remodeling may improve immune cell infiltration, reinforcing the idea that TAMs are not passive players but pivotal therapeutic targets in PDAC [123].

#### Nanotechnology delivery of innate immune agonists

NP-based delivery of innate immune agonists such as STING ligands and TLR agonists has emerged as a promising strategy to reprogram the PDAC immune landscape and enhance antitumor immunity. These agonists stimulate type I interferon (IFN) production, activate DCs, repolarize macrophages, reduce Tregs, and prime cytotoxic lymphocytes. However, systemic administration of free agonists suffers from poor pharmacokinetics and off-target toxicity, while direct injection into pancreatic tumors remains clinically impractical.

NP platforms offer a targeted and safe delivery approach. Chibaya et al. developed ~ 40 nm PEGylated lipid-based immuno-NPs that co-encapsulate a STING agonist (cyclic di-GMP) and a TLR4 agonist monophosphoryl lipid A (MPLA), enabling efficient accumulation in the tumor microenvironment and preferential uptake by tumor-associated APCs [124]. These NPs localized predominantly to myeloid cells and elicited robust IFN-β production, improved antigen presentation, and activated both CD8⁺ T cells and NK cells without inducing systemic toxicity. Similarly, Luo et al. formulated a lipid-coated silica NP (“silicasome”) that co-delivers a TLR7/8 agonist (3 M-052) and irinotecan, achieving localized DC activation, enhanced CD8⁺ T cell infiltration, and a favorable CD8⁺:Treg ratio in PDAC models [125].

Co-formulation of innate immune agonists within the same nanocarrier allows for synchronized immune activation. In preclinical PDAC models, dual delivery of STING and TLR4 agonists via immuno-NPs triggered superior type I IFN signaling, enhanced antigen presentation, and increased infiltration of cytotoxic lymphocytes compared to single-agent or free-drug controls [124]. These effects translated into substantial tumor regression, prolonged survival, and even metastasis eradication. Mechanistically, these responses were dependent on intact STING signaling in both tumor and host cells, as well as functional IFNAR signaling to sustain cytotoxic lymphocyte activity.

Compared to free agonists, NP-mediated delivery improves pharmacokinetics, enhances tumor targeting, and minimizes systemic inflammation. This targeted approach reduces the risk of immune-related toxicities and enables co-delivery of synergistic agents. However, STING agonists have shown limited efficacy in clinical trials, largely due to intrinsic limitations of the STING pathway in humans. Many tumors exhibit suppressed STING signaling through downregulation or loss of cGAS/STING expression, or expression of nonfunctional splice variants, dampening the immunostimulatory response to agonists. Additionally, prevalent human STING polymorphisms—such as the H232 allele—display reduced signaling capacity, rendering standard agonists less effective in certain patient populations. STING activation may also initiate immunoregulatory feedback mechanisms, including autophagy and inflammasome activation, that constrain sustained antitumor immunity [126]. Moreover, immune exhaustion, marked by PD-1 upregulation on T and NK cells during prolonged stimulation, may limit therapeutic durability and necessitate combination with checkpoint inhibitors. Heterogeneous NP distribution within the fibrotic PDAC stroma and nonspecific uptake by non-target tissues further complicate therapeutic efficacy. To address these challenges, strategies such as tumor-responsive NP release, localized delivery, and integration with other immune agonists are being explored.

These findings support the clinical translation of NP-based innate immune agonist therapy. Given the correlation between STING/TLR pathway activity and cytotoxic immune cell presence in human PDAC, this approach holds promise for converting immune-desert tumors into immunologically responsive ones. As early-phase trials emerge, NP-enabled innate immune activation offers a compelling path forward for overcoming immune resistance in PDAC.

### Nanotechnology for modulating desmoplastic stroma and abnormal vasculature in PDAC

The desmoplastic stroma is a defining feature of PDAC, consisting of a dense collagen-rich ECM produced largely by CAFs. This stromal barrier not only impedes drug delivery and immune cell infiltration but also promotes tumor survival and therapy resistance. Advances in nanotechnology have opened new strategies to target and remodel the stroma, thereby enhancing therapeutic penetration and alleviating immunosuppression. In parallel, tumor vasculature in PDAC is highly abnormal (compressed, leaky, and poorly perfused), contributing to hypoxia and inefficient drug delivery. Nanotechnology-based approaches are being explored to normalize tumor blood vessels or exploit their properties for improved NP delivery. By concurrently modulating stroma and vasculature, NPs offer a way to break through multiple physical barriers in PDAC and improve treatment outcomes (Fig. 2D).

#### Nanoparticle-based targeting and modulation of the desmoplastic stroma

CAFs are the principal architects of fibrotic stroma in PDAC, producing excessive ECM components (collagens, fibronectin, HA) that fortify the immunosuppressive in tumor microenvironment. NP-based therapies can target CAFs or their fibrogenic signaling pathways to soften the stroma and enhance drug access. For example, Yu et al. designed a thermosensitive liposome system (HSA-BMS@CAP-ITSL) that releases a small-molecule PD-1/PD-L1 inhibitor (BMS-202) in response to conditions found near CAFs [127]. This complex NP included a peptide (CAP) targeting FAP-α on CAFs, ensuring the liposomes preferentially accumulate in CAF-rich areas​. Upon near-infrared irradiation, the liposomes heat up and release BMS-202, while also inducing a photothermal effect that ablates some stromal tissue and relieves hypoxia. In PDAC models, this dual-action approach suppressed tumor growth and metastasis, likely by deactivating CAFs and simultaneously reactivating local T cells via PD-1/PD-L1 blockade.

Another strategy is to target surface markers on CAFs to deliver drugs that reprogram or kill them. HA-coated cationic albumin NPs have been used to carry an IDO inhibitor plus celastrol (a natural anti-fibrotic agent) directly to CD44-expressing fibroblasts, resulting in reduced IDO-mediated immunosuppression and enhanced T-cell infiltration [128]. This illustrates a general principle: directing nanocarriers to CAFs can modulate the pro-fibrotic and immunosuppressive activity and make the stroma more permissive for immune cells and chemotherapy.

Disrupting key fibrogenic signaling pathways represents a promising strategy to modulate the desmoplastic stroma and enhance therapeutic outcomes in PDAC. Among these, TGF-β is recognized as a master regulator of fibrosis, driving the activation of CAFs, ECM deposition, and immune suppression. To target this pathway, Feng et al. developed CREKA peptide-functionalized PEG-PLA NPs (NPs) encapsulating α-mangostin, a natural small-molecule inhibitor of TGF-β signaling [129]. The CREKA peptide, which selectively binds to fibrin-fibronectin complexes abundant in tumor vasculature and fibrotic stroma, enabled the NPs to home to fibronectin-rich regions within the tumor microenvironment. This targeted accumulation allowed for localized delivery of α-mangostin, resulting in efficient inhibition of TGF-β signaling in CAFs. Consequently, the treatment downregulated fibrotic gene expression, reduced collagen I and fibronectin deposition, and alleviated the dense stromal barrier characteristic of PDAC. When used in combination with gemcitabine, the standard chemotherapeutic for PDAC, the anti-fibrotic NPs significantly enhanced drug penetration and intratumoral accumulation.

Direct ECM degradation or modification is also being pursued carefully. PEGylated human hyaluronidase (PEGPH20) can enzymatically digest HA in PDAC stroma, temporarily decompressing blood vessels and enhancing drug delivery and T-cell entry [130]. To improve tumor-specific delivery and reduce systemic toxicity, a dextran-modified hyaluronidase (DEX–HAase) NP has been developed, utilizing a pH-responsive, traceless linker that ensures enzyme release specifically within the acidic tumor microenvironment. This strategy not only loosens the ECM to facilitate oxygen and drug penetration but also enhances photodynamic therapy (PDT) efficacy and reverses immunosuppression, ultimately improving anti–PD-L1 checkpoint blockade and inducing abscopal effects [131]. Similarly, an oncolytic vaccinia virus encoding hyaluronidase (OVV–Hyal1) has demonstrated robust ECM remodeling, improved drug and immune cell infiltration, and superior antitumor activity when combined with chemotherapeutics, ICIs, or CAR-T cells [132]. One must exercise caution with stromal depletion, however, as excessive ablation of stroma (especially collagen) can lead to tumor collapse and more aggressive invasion if not properly controlled. To mitigate risks, implantable NP-delivery devices or triggered-release systems are under development to confine stromal modulation to the tumor site and avoid systemic effects [99].

In addition to TGF-β inhibition and enzymatic matrix degradation, NP-mediated RNA interference (RNAi) has emerged as a promising strategy for remodeling the desmoplastic stroma in PDAC. Xie et al. engineered a cholesterol-modified polymeric NP bearing a CXCR4 antagonist for the codelivery of anti–miR-210 and KRAS^G12D^ siRNA, designed to simultaneously inactivate PSCs and suppress tumor cell proliferation [133]​. Silencing of miR-210 reprogrammed activated PSCs toward a quiescent phenotype, as evidenced by the downregulation of fibrotic markers such as αSMA and collagen I. Concurrently, CXCR4 inhibition disrupted tumor–stroma interactions, synergistically depleting fibrotic stroma and alleviating local immunosuppression​. This combinatorial strategy led to reduced ECM density, enhanced CD8⁺ T cell infiltration, suppression of metastases, and prolonged survival in orthotopic PDAC models​. In a parallel approach, Zhang et al. developed a “dual-warhead” nanomedicine coated with tumor cell membrane vesicles that co-delivers a Hedgehog (Hh) pathway inhibitor and siRNA targeting SOX9, a transcription factor implicated in stromal fibrosis and immune suppression​ [134]. In this system, the Hh inhibitor suppressed PSC activation and collagen I production, while SOX9 silencing disrupted the SOX9–CXCL5 axis in tumor cells, thereby reducing the recruitment of immunosuppressive MDSCs​. By concurrently dismantling the physical ECM barrier and the immunologic “soft” barrier, this strategy promoted a more immune-permissive microenvironment with increased intratumoral CD8⁺ T cell infiltration, ultimately enhancing the efficacy of T cell–based immunotherapy​. Collectively, these RNAi-based nanotherapies demonstrated potent stromal reprogramming, immune activation, and superior anti-tumor efficacy in preclinical PDAC models, supporting their potential for rational combination with immune checkpoint blockade​​.

Targeting the stroma in PDAC remains significant challenges. While the fibrotic stroma impedes therapy, it also restrains tumor growth; excessive depletion—as seen in trials with Hedgehog pathway inhibitors—can paradoxically accelerate disease progression [135]. Thus, achieving the right degree of stromal modulation is essential, yet complicated by interpatient variability and the heterogeneity of CAFs and ECM components. Single-target approaches (e.g., TGF-β inhibition) may leave compensatory fibrotic pathways intact, while large NPs may struggle to penetrate dense stroma, limiting their efficacy. Sequential or multi-stage NP delivery could improve access but adds complexity. Off-target effects also pose a risk, especially when targeting proteins like FAP, which are expressed in normal fibroblasts involved in wound healing [136]. Enzymatic degradation strategies (e.g., hyaluronidase or collagenase) must be tightly localized—ideally via tumor-specific triggers—to avoid damaging healthy connective tissue. Moreover, the benefits of stromal normalization, such as improved perfusion or T cell infiltration, may be transient, requiring precisely timed combination regimens. Monitoring therapeutic impact is also difficult; current imaging lacks sensitivity for detecting stromal remodeling, necessitating the development of specialized MRI or PET tracers. Altogether, while stromal targeting remains a promising avenue, its clinical success will depend on nuanced, tumor-specific strategies that balance efficacy with safety.

Effective stromal targeting in PDAC requires balance: while reducing fibrosis can enhance drug and immune cell infiltration, complete stromal ablation may remove structural constraints on tumor growth and promote invasion. Nanotechnology enables precise modulation of the stroma, allowing for selective remodeling rather than total depletion. For example, NPs can deliver agents such as vitamin D analogues or all-trans retinoic acid to reprogram CAFs toward a quiescent state, reducing ECM deposition and immunosuppressive signaling while preserving stromal integrity [137]. As our understanding of CAF heterogeneity advances, targeted delivery to specific subtypes—such as FAP⁺ myofibroblasts or IL-6–producing inflammatory CAFs—using ligands for LRRC15 or GPR77 may further refine therapeutic precision [25, 138]. On the matrix side, enzyme-loaded NPs are being engineered for tumor-specific activation—for instance, collagenase coated with an inhibitor that is cleaved by tumor-associated proteases, confining ECM degradation to the tumor site [139]. Combining stromal modulation with immune activation within a single NP—such as “nano-sapper” systems that co-deliver anti-fibrotic agents and T cell–recruiting chemokines—has shown promise in breaking physical and immunologic barriers simultaneously [140]. Emerging technologies like ultrasound-mediated stromal disruption and magnetically guided NPs may enhance intratumoral penetration [141]. While clinical translation remains early, stromal-targeting nanomedicines are poised to complement chemotherapy and immunotherapy by “priming” the tumor for deeper immune and drug access. Ultimately, nanotechnology is shifting stroma targeting from brute-force disruption toward sophisticated reprogramming—transforming fibrotic shield of PDAC into a more permissive, therapeutically responsive microenvironment.

#### Nanotechnology-enabled targeting and normalization of aberrant vasculature

PDAC tumors typically have a dysfunctional vasculature: blood vessels are sparse, irregular, and often collapsed due to high stromal pressure. This leads to tumor hypoxia and prevents immune cells and drugs from adequately perfusing the tumor. Two broad approaches exist to tackle this issue: anti-angiogenesis (targeting pro-angiogenic signals to prevent abnormal vessel growth) and vascular normalization (modulating vessels to make them more functional and less leaky). Nanotechnology plays a role in both, by delivering anti-angiogenic compounds specifically to tumors and by taking advantage of (or fixing) the unique vascular properties to improve therapy delivery.

An example of vascular modulation is given by Zhao et al., who developed polymeric micelles co-loaded with cyclopamine (a Sonic Hedgehog (Shh) pathway inhibitor with anti-angiogenic effects) and PTX (chemotherapy) [142]. Cyclopamine helps reduce tumor angiogenesis and also depletes stromal content, which in turn promotes T cell infiltration into the tumor microenvironment. When these micelles (M-CPA/PTX) were combined with anti-PD-1 checkpoint blockade, they increased intratumoral CD8⁺ T cell infiltration and sensitized tumors to the checkpoint inhibitor, yielding improved survival in a PDAC model​. The NP delivered cyclopamine directly to the tumor, maximizing its local effect on vasculature and stroma while minimizing systemic toxicity.

Another innovative strategy involved a “Nano-sapper” system combining α-mangostin phosphate (a vascular-normalizing agent) and LIGHT cytokine (to stimulate immune cells) in a NP that targeted the tumor vasculature [140]. This treatment increased VE-cadherin expression on endothelial cells and reduced vessel permeability, effectively normalizing the blood vessels. The normalized vasculature improved oxygenation and allowed greater infiltration of cytotoxic T lymphocytes, thereby turning the previously immune-excluded tumor into one more susceptible to immune attack​. Despite progress, challenges remain in controlling optimal vessel normalization window. One issue is the transient nature of the normalization window—there is often a limited time after treatment during which vessels are more perfused and permeable to immune cells or drugs. NPs might help here by providing sustained release of normalizing agents or by acting within that window to deliver their cargo. Additionally, the impact of normalized vessels on NP delivery needs further study; more perfused vessels might increase NPs penetration, but less leaky vessels could reduce NP extravasation. Therefore, designing NPs of appropriate size and surface properties with controlled release capabilities is important to ensure they still extravasate after vessels tighten up and efficiently release therapeutic cargoes.

Nanotechnology-based vascular modulation in PDAC, though promising, faces significant challenges. Vessel normalization is inherently transient—its therapeutic window is narrow and dose-dependent, and suboptimal timing or excessive anti-angiogenesis may close the window prematurely or worsen hypoxia [143]. The hypovascular and high-pressure microenvironment in PDAC further limits uniform NP distribution, even after normalization, due to variability in vessel architecture and stromal density. Moreover, the disorganized vasculature that impedes delivery is also exploited by many nanotherapies via the enhanced permeability and retention (EPR) effect; thus, normalization can paradoxically reduce extravasation of larger NPs. For instance, ~ 20–30 nm particles may benefit, while ~ 100 nm NPs show reduced uptake post-normalization [144]. Precise coordination of drug release within this window is technically demanding. Collectively, these limitations highlight the need for refined strategies to fully realize the potential of vascular-targeted nanotherapies in PDAC.

To overcome current limitations, future vascular-targeting nanotherapies in PDAC are increasingly focused on precision delivery and real-time guidance. Smart or stimuli-responsive NPs that release their payload in response to tumor-specific cues—such as acidic pH, hypoxia, or enzyme activity—offer controlled release synchronized with optimal vascular perfusion. Concurrently, the integration of advanced imaging modalities (e.g., MRI, photoacoustic imaging) and computational modeling is developing for real-time tracking of vascular normalization and NP transport, allowing clinicians to personalize treatment timing for maximal uptake [145, 146]. Beyond directly modulating vessels, nanotechnology can also exploit the chaotic vasculature of tumors via the EPR effect to accumulate drugs in tumors. However, in PDAC the EPR effect is less pronounced due to poor perfusion. Thus, a hybrid approach of partially normalizing vessels (to get more blood flow) but still allowing some leakiness (to let NPs out) could be ideal. Future research is focusing on improving the precision of dosing and timing for anti-angiogenic therapies to maintain a beneficial level of vessel normalization, and on combining these with nanocarriers for other therapies (e.g., delivering T cell–attracting chemokines or additional drugs during the normalization window). By synergizing vascular normalization with nanomedicine, we can maximize therapeutic delivery and immune cell entry to the tumor while the vessels are in a favorable state.

In conclusion, combinatorial nanotherapy strategies targeting both the stroma and vasculature offer a comprehensive strategy to overcome physical barriers of PDAC. These approaches are not only relevant to PDAC but could extend to other solid tumors with similar challenges (dense stroma, poor vasculature, and immune exclusion). Moreover, the versatility of nanotechnology allows integration with other modalities like photothermal therapy, ultrasound (for enhancing NP delivery), or even oncolytic virotherapy, creating multi-modal treatments for treating PDAC.

## Clinical translation and ongoing trials

Translating nanomedicine-based immunotherapies for PDAC into clinical success remains a formidable challenge. Nevertheless, several clinical trials are underway or have recently concluded, exploring combinations of nanotechnology with immunotherapy (Table 3). These studies provide insight into which strategies show promise and which obstacles remain.

**Table 3:** Current clinical trials of nanomedicine-based immunotherapies in PDAC

Intervention (Clinical trial)	Formulation/Nanomedicine aspect	Phase	Status	Primary outcome:	Secondary outcome:	Brief description and outcome	Ref
**mRNA-5671/V941 (G12D**,** G12V**,** G13D**,** and G12C) + Pembrolizumab** - mRNA- vaccine in combination with pembrolizumab (NCT03948763)	Lipid NP-based mRNA vaccine (KRAS mutation); anti–PD-1 antibody (pembrolizumab)	Phase I	Completed	Assessing the dose-limiting toxicity and number of participants experienced an adverse event	Evaluating the objective response rate (ORR) and presence of mutant KRAS specific t cells	The combination regimen induced minimal KRAS-specific T cell responses; serious adverse events and the low objective response rate (ORR) warrant further evaluation.	[62]
**Autogene cevumeran (BNT122) + atezolizumab**– personalized mRNA neoantigen vaccine given after surgery (NCT04161755)	mRNA–lipoplex NP vaccine (patient-specific tumor neoantigens), combined with anti–PD-L1 antibody (atezolizumab) and chemotherapy (FOLFIRINOX)	Phase I	Completed; Phase II ongoing (adjuvant setting)​	Evaluating the safety of a personalized tumor vaccine combined with atezolizumab and mFOLFIRINOX	N/A	In 16 resected PDAC patients, it was safe and immunogenic: ~50% of patients developed robust T cell responses against neoantigens, correlating with prolonged disease-free survival​. A Phase II trial is underway to evaluate this nanovaccine as an adjunct to standard therapy.	[53] [147]
**BL-8040 + pembrolizumab + liposomal irinotecan (Onivyde**^®^)– **multi-agent chemo-immunotherapy (NCT02826486)​**	CXCR4 inhibitor (BL-8040) mobilizer; NP liposomal irinotecan (PEGylated liposome) for tumor-targeted chemotherapy; anti–PD-1 antibody (pembrolizumab)	Phase II ​	Completed	Assessing ORR by target lesions identified in CT or MRI imaging according to RECIST 1.1 Criteria	Assessing overall survival, progression-free survival (pfs), and disease control (sum of partial response (pr), complete response (cr) and stable disease (sd).) by imaging (RECIST 1.1)	The regimen showed increased immune cell infiltration and was well-tolerated, but only limited clinical responses, underscoring the need for multi-modal combination strategies​.	[109]
**Oncolytic IL-12 adenovirus (Ad) therapy**– intratumoral cytokine virus immunotherapy (NCT03281382)​	Engineered oncolytic adenovirus encoding human IL-12 (no synthetic NP, but virus serves as a biological nanocarrier)	Phase I ​	Completed	Evaluating the toxicity of the gene therapy	Reporting grade 3 adverse events	The Phase I trial demonstrated localized immune activation (increased intratumoral IFN-γ and CD8⁺ T cells) but minimal systemic effects.	[151]
**CNSI-Fe(II) ferroptosis NP**– novel injectable ferroptosis inducer for immune priming (NCT06048367)​	Carbon-based NP loaded with ferrous iron (CNSI-Fe(II)) to catalyze intra-tumoral oxidative damage (ferroptotic cell death) and disrupt dense stroma	Phase I ​	Recruiting (advanced KRAS-mutant PDAC)	Assessing the safety and tolerability of CNSI-Fe(II), including Dose-Limiting Toxicity (DLT) and Maximum Tolerated Dose (MTD)	Assessing the pharmacokinetic (PK) profile of CNSI-Fe(II)	The trial will evaluate safety, immune effects, and preliminary efficacy of this approach.	[152]
**NBTXR3 + radiotherapy**– hafnium oxide NP radio-enhancer activated by radiation (NCT03589339)	Inorganic radioenhancer NP (NBTXR3) injected into tumor; amplifies local radiotherapy effects (increased DNA damage and tumor kill)	Phase I	Completed (locally advanced PDAC)	Determinating the recommended dose and the safety evaluation at RP2D	Evaluating the anti-tumor response of R3/RT/PD-1; Assessing the safety and feasibility of R3/RT/PD-1; Evaluating the body kinetic profile of intratumorally injected NBTXR3	In Phase I, NBTXR3 was safely injected into pancreatic tumors.	[153]
**Neoadjuvant gemcitabine + nab-paclitaxel + durvalumab + oleclumab**– chemo plus dual immunotherapy before surgery (NCT04940286)​	NP albumin-bound paclitaxel (nab-PTX, Abraxane^®^) chemotherapy; anti–PD-L1 antibody (durvalumab) and anti-CD73 antibody (oleclumab) targeting adenosine pathway	Phase II ​	Active, not recruiting (neoadjuvant, resectable PDAC)	Assessing the major pathological response rate and incidence of adverse events	Evaluating radiographic response rate, recurrence-free survival, overall survival, frequency of intraoperative and postoperative complications; Changing in circulating tumor deoxyribonucleic acid (ctDNA) levels	Trial will assess pathologic response and survival benefit of this combination immune-nanotherapy.	[154]

### Nanoparticle vaccines driving tumor-specific immunity in PDAC

Therapeutic cancer vaccines formulated with nanocarriers are showing promise in PDAC by eliciting tumor-specific T cell responses. A Phase I trial of autogene cevumeran (BNT122), a personalized mRNA-lipoplex vaccine encoding patient-specific neoantigens, demonstrated safety and immunogenicity in 16 PDAC patients receiving surgery, chemotherapy, and anti–PD-L1 therapy [63, 147]. About half of the patients mounted robust neoantigen-specific T cell responses, and 8 remained relapse-free at 18 months. Follow-up revealed that 6 of these 8 patients were still disease-free at 3 years, with long-lived CD8⁺ T cells persisting up to 4 years, suggesting durable immunological memory. This maintenance of immunity points to the potential of nanovaccines to convert PDAC into a chronic, manageable disease. A Phase II trial is now underway to assess its benefit as an adjuvant to standard therapy.

Building on this, investigators are evaluating personalized nanovaccines as maintenance therapy. In a case study, an advanced PDAC patient who had failed multiple therapies received a 12-peptide neoantigen nanovaccine with anti–PD-1 checkpoint inhibition. The patient developed T cell responses against 9 neoantigens, exhibited strong interferon-γ activity, and achieved disease control lasting 10.5 months. Functional neoantigen-specific T cells remained detectable throughout treatment. This case underscores how sustained vaccination can synergize with checkpoint blockade to re-engage the immune system, even in refractory metastatic PDAC. Maintenance vaccine strategies are now being incorporated into trial designs to delay relapse and prolong survival [148].

Other innovative vaccine approaches are being explored. Off-the-shelf nanovaccines targeting shared PDAC antigens, such as KRAS and MUC1, are in development. One preclinical study demonstrated a liver-targeted mRNA nanovaccine capable of reprogramming the immunosuppressive liver microenvironment to attack metastatic PDAC cells [65]. The formulation, which delivered both antigen and immune adjuvants to the liver, reduced tumor burden and prevented new metastases in mice, while generating memory T cells that protected against recurrence. Although preclinical, this work exemplifies how organ-targeted nanocarriers might activate immunity in otherwise immune-privileged metastatic sites. Collectively, these early efforts position NP vaccines—particularly personalized mRNA formulations—as a promising strategy to overcome PDAC’s low immunogenicity.

In brief, a Phase I trial of a personalized mRNA–lipoplex nanovaccine (autogene cevumeran) demonstrated safety and robust neoantigen-specific T cell responses in PDAC patients, with durable disease control in responders. Preclinical studies further support these findings, showing that lipid or polymer-based NPs delivering KRAS-mutant mRNA, tumor lysates, or neoantigens can enhance dendritic cell activation and cytotoxic T cell responses, reduce tumor burden, and improve survival in PDAC models. These studies highlight nanovaccines as a clinically actionable strategy and support their continued development for overcoming immune resistance in PDAC.

### Cytokine and gene delivery via nanocarriers

Nanocarriers are also enabling the targeted delivery of cytokines and immune-modulating genes directly into the PDAC tumor microenvironment. A recent preclinical study showed that lipid NP–delivered IL-12 mRNA, combined with stereotactic radiation, reprogrammed exhausted T cells into highly proliferative effectors, repolarized suppressive myeloid cells, and improved tumor control [149]. These findings support ongoing trials testing intratumoral cytokine mRNA lipid NPs (mRNA-LNPs) in PDAC. Additionally, a triple cytokine mRNA cocktail (OX40L, IL-23, IL-36γ) delivered via NPs is currently being evaluated in a Phase I trial for solid tumors (NCT03739931). Supported by promising preclinical data, this strategy may hold potential for future testing in PDAC [150]. These approaches aim to stimulate local immune activity while minimizing systemic toxicity—a critical balance in cytokine-based therapy.

Though not a classical NP, oncolytic viruses carrying immune cytokines (ex: IL-12 or GM-CSF) have been utilized in PDAC to stimulate immunity. A Phase I using an oncolytic adenovirus encoding IL-12 reported local immune effects but limited systemic impact​ (NCT03281382) [151]. Researchers are now investigating whether combining oncolytic virotherapy with nanocarriers. For example, loading viruses into biodegradable polymer matrices or tagging with NPs for better tumor targeting, leading to enhanced delivery and antitumor immunity.

### Modulating the PDAC stroma with nanotechnology

A major objective of nano-immunotherapy in PDAC is to remodel the dense, fibrotic stroma and immunosuppressive tumor microenvironment, which collectively hinder both immune cell infiltration and therapeutic delivery. One novel approach under investigation is CNSI-Fe(II), a ferroptosis-inducing carbon NP currently in a Phase I trial (NCT06048367) [152]. Designed for intratumoral injection in KRAS-mutant PDAC, this nanoplatform delivers ferrous iron to catalyze oxidative tumor cell death and stromal disruption, potentially releasing tumor antigens and priming an immune response.

Beyond biochemical reprogramming, nanotechnology is also being used to physically modulate the PDAC stroma. NBTXR3, a hafnium oxide NP, enhances radiotherapy-induced DNA damage and tumor cell death. In a Phase I trial, it was safely injected into locally advanced PDAC tumors and activated by radiation, potentially acting as an in situ vaccine by promoting antigen release [153]. Similarly, gold nanoshell-based photothermal therapy is being explored to locally ablate tumors and disrupt stroma through heat-induced ICD. While still experimental in PDAC, these modalities have shown promise in other solid tumors and could be combined with immunotherapies to further improve immune infiltration and therapeutic response.

Nanotechnology is also facilitating combination chemoimmunotherapy strategies aimed at remodeling the tumor microenvironment. A Phase II trial (NCT02826486) tested a multi-agent regimen combining liposomal irinotecan (Onivyde^®^), the CXCR4 inhibitor BL-8040, and pembrolizumab. Here, the nanocarrier enhanced irinotecan delivery while BL-8040 was intended to overcome immune cell exclusion by disrupting the CXCL12-CXCR4 axis. Although clinical responses were limited, the study demonstrated the feasibility and rationale of integrating nanomedicine into immunotherapy regimens [109]. Another ongoing Phase II trial (NCT04940286) is evaluating neoadjuvant gemcitabine and NP nab-PTX with dual checkpoint and adenosine pathway blockade (durvalumab and oleclumab) in resectable PDAC [154]. Nab-PTX not only delivers cytotoxic payloads efficiently but may also enhance stromal penetration, thereby potentiating immune checkpoint responses. These studies reflect a broader shift toward multi-modal strategies that use nanotechnology to remodel the stroma and support more effective immunotherapeutic intervention in PDAC.

Despite promising early-phase results, including prolonged recurrence-free survival (RFS) and robust immune activation in select patients, translating nanotechnology-based immunotherapies into routine PDAC treatment remains challenging. A major barrier is the profoundly immunosuppressive tumor microenvironment, which restricts both immune cell function and therapeutic efficacy—particularly in advanced-stage patients. Additionally, variability in tumor biology, stromal composition, and immune phenotypes complicates patient stratification. The lack of predictive biomarkers and standardized criteria for assessing response to immunotherapy limits trial design and data interpretation. Moreover, manufacturing, scalability, and regulatory complexity associated with personalized nanomedicines—such as individualized mRNA vaccines—pose logistical hurdles for broad clinical implementation. These limitations underscore the need for larger, biomarker-informed trials and improved delivery strategies that can navigate the biological complexity of PDAC.

## Conclusions and future perspectives

Despite major advances in cancer immunotherapy, PDAC remains one of the most treatment-resistant solid tumors. Its characteristically dense desmoplastic stroma, poor immune infiltration, and profoundly immunosuppressive tumor microenvironment create a hostile environment for both immune cells and therapeutics. Nanotechnology has emerged as a versatile tool to overcome many of these barriers, offering targeted delivery, enhanced drug retention, and co-delivery of immunomodulatory agents. While recent preclinical and early clinical studies highlight the promise of nanomedicine-based immunotherapy in PDAC, substantial translational challenges remain.

One major limitation lies in the preclinical models currently used to evaluate these therapies. Genetically engineered mouse models (GEMMs) and patient-derived xenografts (PDXs) have been instrumental in advancing our understanding of PDAC biology but still fall short in fully recapitulating the complexity of human disease—particularly the heterogeneity of the tumor microenvironment, the dynamic immune-stromal interactions, and the mechanisms of immune evasion and metastasis [155]. These models often fail to capture the fibrotic density and immunosuppressive signaling networks found in human PDAC [156]. To bridge this gap, the development of more predictive platforms—such as humanized mouse models or advanced organoids incorporating human immune and stromal components—is urgently needed. Such systems will be invaluable for evaluating how NPs penetrate the fibrotic ECM, interact with immune cells, and modulate immunotherapy responses in a more clinically relevant context.

Another critical hurdle is the immunogenicity of nanocarriers themselves. For example, PEGylated NPs, widely used to extend circulation half-life and reduce nonspecific clearance, can provoke the formation of anti-PEG antibodies in some patients. These antibodies may lead to accelerated blood clearance (the ABC phenomenon) and hypersensitivity reactions (HSRs) upon repeated dosing—undermining the safety and efficacy of NP-based treatments [69, 157]. This is particularly problematic in PDAC patients, who often require multiple cycles of therapy over extended periods. To overcome this, researchers are exploring alternative “stealth” strategies, such as zwitterionic coatings or biomimetic surface modifications that reduce recognition by the immune system. Another approach involves co-delivery of transient immunosuppressive agents to blunt anti-NP immune responses during early dosing cycles [158]. Continued innovation in surface engineering will be essential to preserve the pharmacokinetic advantages of PEGylation while minimizing immune-related liabilities.

The fibrotic stroma in PDAC remains a double-edged sword. While it clearly restricts the intratumoral diffusion of therapeutics—including NPs—it also plays a complex role in tumor biology. Total ablation of the stroma has, in some cases, resulted in accelerated tumor progression and metastasis, underscoring the need for selective modulation rather than complete removal [159, 160]. NP design must therefore evolve to address this delicate balance. Future formulations may incorporate features such as ultra-small size, flexible or shape-shifting structures, and stress-responsive materials that adapt to the tumor’s physical constraints. For instance, filamentous or deformable NPs may better navigate through collagen-rich regions, while tumor-penetrating peptides displayed on NP surfaces can facilitate deep tissue infiltration by interacting with receptors in the tumor interstitium [161]. In addition, localized delivery strategies—such as NP-releasing implants placed directly into the tumor bed or peritoneal cavity—offer an opportunity to bypass systemic circulation entirely and deposit nanotherapies precisely where they are most needed, increasing local drug concentration and therapeutic penetration [6, 106].

In conclusion, nanotechnology offers a powerful avenue for overcoming many of the immunological and physical barriers that have limited progress in PDAC immunotherapy. By improving the pharmacokinetics of immunotherapeutics, shielding them from degradation, modulating the tumor microenvironment, and enabling combination delivery strategies, nanomedicine has the potential to convert PDAC from an immune “desert” into a more immunologically active tumor.

Among the various nanotechnology platforms explored in this review, mRNA vaccines have shown the most promising translational potential for immunotherapy in PDAC, as evidenced by both preclinical data and encouraging results from early-phase clinical trials. Personalized mRNA–lipoplex nanovaccines encoding neoantigens have induced robust and durable T cell responses, with some patients achieving long-term relapse-free survival. These findings position mRNA vaccines as a frontrunner in the nanomedicine-enabled immunotherapy landscape for PDAC. Looking forward, the development of nanocarriers capable of efficient, nucleus-targeted DNA delivery—with sustained expression, minimal immunogenicity, and precise spatiotemporal control—may open new avenues for cancer immuno-gene modulation strategies. Such advances could significantly expand the therapeutic repertoire beyond current mRNA-based systems and further enhance the immunogenic reprogramming of the PDAC microenvironment. However, to achieve broader clinical success, the field must continue to tackle key challenges: building better disease models, mitigating NP-induced immune reactions, and optimizing NP penetration into the tumor core.

Future efforts should prioritize rational NP design, incorporating smart materials, multi-functional targeting, and responsive release systems. In parallel, a deeper understanding of PDAC immunobiology—including patient-specific immune profiles and stromal interactions—will be crucial to guide the design of personalized nanomedicine approaches. Ultimately, integrating nanotechnology with immunotherapy offers a transformative path forward. By dismantling the layered immunosuppressive defenses of PDAC, such strategies hold the potential to improve patient outcomes and deliver meaningful survival benefits in PDAC.

## Data Availability

Not applicable.

## Abbreviations

ABC: Accelerated blood clearance
APCs: Antigen-presenting cells
apCAFs: Antigen-presenting fibroblasts
aPSCs: Activated pancreatic stellate cells
α-SMA: α-Smooth muscle actin
Bregs: Regulatory B cells
CAF: Cancer-associated fibroblast
CDCs: Conventional DCs
DAMPs: Damage-associated molecular patterns
DCs: Dendritic cells
ECM: Extracellular matrix
ER: Endoplasmic reticulum
ERS: Endoplasmic reticulum stress
EPR effect: Enhanced permeability and retention effect
FAP-α: Fibroblast activation protein-α
GEMMs: Genetically engineered mouse models
GFAP: Glial fibrillary acidic protein
GM-CSF: Granulocyte-macrophage colony-stimulating factor
HA: Hyaluronic acid
HLA: Human leukocyte antigen
HSCs: Hematopoietic stem cells
HSRs: Hypersensitivity reactions
iCAFs: Inflammatory cancer-associated fibroblasts
ICD: Immunogenic cell death
ICIs: Immune checkpoint inhibitors
IDO: Indoleamine 2,3-dioxygenase
IrAEs: Immune-related adverse effects
IPMN: Intraductal papillary mucinous neoplasms
LIF: Leukemia inhibitory factor
LNPs: Lipid nanoparticles
mFOLFIRINOX: Modified FOLFIRINOX
MRI: Magnetic resonance imaging
MCN: Mucinous cystic neoplasms
MDSC: Myeloid-derived suppressor cells
MMPs: Matrix metalloproteinases
MPLA: Monophosphoryl lipid A
mtDNA: Mitochondrial DNA
myCAFs: Myofibroblastic cancer-associated fibroblast
NK cells: Natural killer cells
NOD-like receptor: Nucleotide-binding oligomerization domain-like receptors
NP: Nanoparticle
ORR: Objective response rate
PanIN: Pancreatic intraepithelial neoplasia
PDAC: Pancreatic ductal adenocarcinoma
PDGF: Platelet-derived growth factor
PDXs: Patient-derived xenografts
PPa: Photosensitizer pyropheophorbide a
PSC: Pancreatic stellate cell
PTX: Paclitaxel
RFS: Recurrence-free survival
ROS: Reactive oxygen species
SASP: Senescence-associated secretory phenotype
Shh: Sonic hedgehog
SLNs: Solid lipid nanoparticles
SPIONs: Superparamagnetic iron oxide nanoparticles
TAAs: Tumor-associated antigens
TAMs: Tumor-associated macrophages
TGF-β: Transforming growth factor-β
TLR: Toll-like receptor
Tregs: Regulatory T cells
TSAs: Tumor-specific antigens
WT1: Wilms tumor 1
